# Association of environmental markers with childhood type 1 diabetes mellitus revealed by a long questionnaire on early life exposures and lifestyle in a case–control study

**DOI:** 10.1186/s12889-016-3690-9

**Published:** 2016-09-29

**Authors:** F. Balazard, S. Le Fur, S. Valtat, A. J. Valleron, P. Bougnères, Isis-Diab collaborative group, Dominique Thevenieau, Corinne Fourmy Chatel, Rachel Desailloud, Hélène Bony-Trifunovic, Pierre-Henri Ducluzeau, Régis Coutant, Sophie Caudrelier, Armelle Pambou, Emmanuelle Dubosclard, Florence Joubert, Philippe Jan, Estelle Marcoux, Anne-Marie Bertrand, Brigitte Mignot, Alfred Penformis, Chantal Stuckens, Régis Piquemal, Pascal Barat, Vincent Rigalleau, Chantal Stheneur, Sylviane Fournier, Véronique Kerlan, Chantal Metz, Anne Fargeot-Espaliat, Yves Reznic, Frédérique Olivier, Iva Gueorguieva, Arnaud Monier, Catherine Radet, Vincent Gajdos, Daniel Terral, Christine Vervel, Djamel Bendifallah, Candace Ben Signor, Daniel Dervaux, Abdelkader Benmahammed, Guy-André Loeuille, Françoise Popelard, Agnès Guillou, Pierre-Yves Benhamou, Jamil Khoury, Jean-Pierre Brossier, Joachim Bassil, Sylvaine Clavel, Bernard Le Luyer, Pierre Bougnères, Françoise Labay, Isabelle Guemas, Jacques Weill, Jean-Pierre Cappoen, Sylvie Nadalon, Anne Lienhardt-Roussie, Anne Paoli, Claudie Kerouedan, Edwige Yollin, Marc Nicolino, Gilbert Simonin, Jacques Cohen, Catherine Atlan, Agnès Tamboura, Hervé Dubourg, Marie-Laure Pignol, Philippe Talon, Stéphanie Jellimann, Lucy Chaillous, Sabine Baron, Marie-Noëlle Bortoluzzi, Elisabeth Baechler, Randa Salet, Ariane Zelinsky-Gurung, Fabienne Dallavale, Etienne Larger, Marie Laloi-Michelin, Jean-François Gautier, Bénédicte Guérin, Laure Oilleau, Laetitia Pantalone, Céline Lukas, Isabelle Guilhem, Marc De Kerdanet, Marie-Claire Wielickzo, Mélanie Priou-Guesdon, Odile Richard, François Kurtz, Norbert Laisney, Déborah Ancelle, Guilhem Parlier, Catherine Boniface, Dominique Paris Bockel, Denis Dufillot, Berthe Razafimahefa, Pierre Gourdy, Pierre Lecomte, Myriam Pepin-Donat, Marie-Emmanuelle Combes-Moukhovsky, Brigitte Zymmermann, Marina Raoulx, Anne Gourdin et Catherine Dumont

**Affiliations:** 1 Sorbonne Universités, UPMC Univ Paris 06, CNRS, Paris, France; 2 INSERM U1169, Hôpital Bicêtre, Université Paris-Sud, Kremlin-Bicêtre, France; 3 Department of pediatric endocrinology, Hôpital Bicêtre, Kremlin-Bicêtre, France

**Keywords:** Case–control, Epidemiology, Type 1 diabetes, Data-driven, Environment

## Abstract

**Background:**

The incidence of childhood type 1 diabetes (T1D) incidence is rising in many countries, supposedly because of changing environmental factors, which are yet largely unknown. The purpose of the study was to unravel environmental markers associated with T1D.

**Methods:**

Cases were children with T1D from the French Isis-Diab cohort. Controls were schoolmates or friends of the patients. Parents were asked to fill a 845-item questionnaire investigating the child’s environment before diagnosis. The analysis took into account the matching between cases and controls. A second analysis used propensity score methods.

**Results:**

We found a negative association of several lifestyle variables, gastroenteritis episodes, dental hygiene, hazelnut cocoa spread consumption, wasp and bee stings with T1D, consumption of vegetables from a farm and death of a pet by old age.

**Conclusions:**

The found statistical association of new environmental markers with T1D calls for replication in other cohorts and investigation of new environmental areas.

**Trial registration:**

Clinical-Trial.gov NCT02212522. Registered August 6, 2014.

**Electronic supplementary material:**

The online version of this article (doi:10.1186/s12889-016-3690-9) contains supplementary material, which is available to authorized users.

## Background

The current rise in T1D incidence [1] is attributed to environmental causes to which genetically predisposed children are increasingly exposed, but epidemiology has delivered more questions than robust answers. Dissecting the environment is a daunting task, with paramount difficulties for extracting relevant information from multiple known and unknown exposures occurring during childhood. The fact that childhood T1D occurs early in life allows restraining the environmental analysis to the few years encompassing intrauterine life, infancy and childhood. A classical way of doing this is using retrospective questionnaires, but the questions are necessarily limited to selected areas of child life and answers may be biased by parental recall. Environmental comparison between cases and controls can also be prospective. To achieve this given the low prevalence of T1D, it is necessary to study a genetically at risk population, for example positivity for HLA screening in the TEDDY study [2]. Another way of avoiding recall-related problems is to use registries [3]. However, registries are more limited in their scope than a questionnaire. Another difficulty inherent to any environmental approach is that participants are not aware of many exposures. Collecting biological samples to characterize the “exposome” [4] of T1D children also has several drawbacks, since blood parameters may be modified as a consequence of T1D not as a causal component, and are confined to the only environmental parameters that leave a long living trace in patient’s blood, i.e. a minority of exposures.

Over the recent years, suspicion has almost exclusively focused on infectious agents and nutrition in the early years of life [5–7]. Enteroviruses have been the subject of numerous studies and have remained the most often suspected environmental contributors to T1D [8, 9]. In contrast, infections have been considered as protective from T1D according to the hygiene hypothesis, which postulates that the increase in autoimmune T1D could be due to the decrease of early infections [10, 11] or lack of parasites [12]. This has been shown in the isogenic NOD mice model [11, 13], but epidemiological evidence in humans, who are exposed to different infectious agents and have a wide genetic variation, is still pending. Studies attempting to relate infectious episodes with T1D have yielded contrasted results [14]. Respiratory infections in the first year of life have been shown to increase the risk of seroconversion to islet autoimmunity (IA) in the BABYDIET cohort and in the MIDIA study [15, 16]. On the other hand, they were not associated with T1D in the DAISY cohort [17]. Gastrointestinal illnesses at precise periods were associated with higher risk of IA in the same study. More recently, the gut microbiome has been investigated in search of a bacterial composition that could be associated with T1D [18].

Nutrition has been the other focus of environmental research for T1D. Overfeeding and the ensuing increase of beta cell functional activity for producing more insulin has been suspected to favor autoimmunity towards the beta cells (the overload hypothesis) [19]. Meta-analyses have found that early weight gain [20] or obesity [21] showed a modest association with T1D. Vitamin D supplementation studied through questionnaires has been suggested to protect from T1D [22], but this has not been confirmed when 25-hydroxyvitamin D levels in plasma were studied [23]. Since vitamin D supplementation of infants is generalized in French infants since the 70s, vitamin D deficiency is not likely to be a driver of increasing T1D incidence.

Several dietary interventions have attempted to prevent T1D. TRIGR tested whether substitution of cow’s milk by casein hydrolysate formula affects the occurrence of IA or progression to T1D [24]. No significant difference has been observed between the two groups for the appearance of IA [25]. Result for the second primary end-point will only be available in 2017. The possibility that exclusive breast-feeding or late introduction to cow’s milk is associated with a modest protection is supported by a meta-analysis of observational studies [26]. A few other nutrients have been studied. An older age at first introduction to gluten showed no protective effect in the BABYDIET study [27]. Omega-3 fatty acids seemed to be associated with a slightly reduced risk of islet autoimmunity in the DAISY cohort [28] but the pilot study that was then performed did not show significant protection [29]. Nicotinamide did not modify the progression to T1D in children with IA in the ENDIT trial [30]. Other prevention trials are underway [31]. Early nutrition is a favorable field of investigation through randomized trials since a vast number of factors can be manipulated experimentally.

The BABYDIAB and the DAISY cohort have found that IA often appears in the first years of life preceding clinical diagnosis T1D by several months or years [32, 33], which stress a potential predisposing role for early environmental exposures. This has inspired our approach for screening early life events that could be associated with environmental differences between cases and controls, including a number of infectious and nutritional exposures that can be reliably recalled by parents.

Our study is a tentative and still limited step for moving environmental research from hypothesis-driven to more data-driven approaches. A comparable move has occurred in the 90s when genetic research has switched its candidate gene approach of complex diseases, notably T1D, to interrogate the complete genome variation blindly with genome wide association studies (GWAS) with the aim of unraveling disease markers [34] that could secondarily lead to true genetic causation [35, 36]. Environment wide association studies (EWAS) [37] or exposome association studies [4] will likely allow researchers to investigate children environment on a vast scale without making a priori hypotheses. Such approaches will remain limited because a myriad of environmental markers will escape investigation, while genomic variation is finite. In this respect, our current 845-item questionnaire can only be viewed as a preliminary proof-of-concept approach for scanning the environment of a child. It is indeed limited by the number of questions that have been selected to describe this environment, by the recall errors that could be made by the parents of the cases and controls, and by the number of participants who agree to spend two hours filling a complex questionnaire. False positivity is an expected weakness of this approach, but careful statistical analysis can provide a list of environmental markers for which false discoveries are controlled.

## Methods

### Questionnaire

The questionnaire was built by a group of academics composed of obstetricians and pediatricians specialized in pediatric infectious diseases, nutrition, and lifestyle. Their task was to define the environment of pregnant women, neonates, infants and young children, by enumerating all aspects that they thought a mother will likely be able to recall years later. A group of mothers of young children (living in urban or rural environment) were also asked to participate. A first questionnaire of nearly 1000 questions was built and tested across 100 young mothers. Only questions that could be answered rapidly were kept, because we considered that the speed of the answers would favor spontaneity and minimize recall errors and bias. The questionnaire was also tested in 30 mothers of young children with recently diagnosed T1D and 30 mothers of children who had declared T1D five to ten years before. Only questions that had a comparable recall score in the two groups of mothers were kept in an effort to eliminate questions that could not be easily recalled. The final questionnaire contained 576 main questions and 845 items when counting sub-questions about the environment including 90 questions about pregnancy, 25 concerning the delivery and early post-natal life, 20 about early childhood, 75 on the subject’s medical life, 60 on nutrition, 40 on housing, 30 on daycare, 30 on leisure and trips, 80 on contact with animals and 60 on family members’ environment. Depending on mothers, the time to fill the questionnaire ranged from 90 to 120 min. A PDF version of it in French is available as Additional file 1.

### Data collection

The Isis-Diab cohort is a large multi-centric cohort of T1D patients in France which recruitment started in 2007. Starting in March 2010, three copies of the questionnaire were sent to the parents of 6618 T1D patients enrolled in the cohort during the month following their inclusion in the study. Parents were asked to fill the questionnaire regarding the exposures and events having taken place in their child’s life before the clinical onset of T1D. They were also asked to enroll as controls two of their friends having an unaffected child of the same age. The 6144 parents having provided a phone number were contacted once during the week following the questionnaire sending. If the questionnaire was not returned within 3 months, parents received a reminder by mail.

One thousand seven hundred sixty-nine cases (i.e. 27 % of the patients to which a questionnaire was sent) and 1085 controls returned the questionnaire. Two hundred forty-one cases provided two controls, 451 cases provided one control, and 1077 cases provided no control. One hundred fifty-two controls were not associated to a case that returned his questionnaire. All the questionnaires completed by patients and controls were seized by a private provider (numerical input for all the « checkbox » responses, and dual manual entry for handwritten responses).

Patients living in areas with higher economic deprivation were less likely to respond [31].

The questionnaire investigated the period preceding diagnosis of the disease. Matched controls were asked to fill the questionnaire with respect to the age at which the patient had been diagnosed. We will refer to this age as the reference age.

### Pre-analysis treatment

A computerized treatment was designed to code categorical questions into binary variables and to allow analysis of sub-questions. After the pre-analysis treatment, 845 variables were available for analysis.

In order for effect sizes to be on a similar scale even though we have binary questions and ordinal questions with up to 5 different levels. For example, consumption of cola drinks frequency was quantified on five levels from never to several times a day. All variables were scaled to be between 0 and 1. In this way, the effect size for ordinal variables corresponds intuitively to the odds ratio between the two extreme responses. The encoding of the variables were modified so that the directionality of the effects be intuitive. A description of the 845 variables is available in Additional file 2.

### Exclusions

We excluded from the analysis the questionnaires where more than 50 % of the questions were left unanswered.

As our questionnaire was designed to quantify a child’s environment, we included only participants whose reference age was between 0.5 years and 15.5 years. To minimize recall errors, we excluded participants for whom the delay between diagnosis and questionnaire reception was greater than 10 years.

We used primary school attendance as another marker of the quality of recall: we excluded participants who reported that their child attended primary school before 5.5 years. In the additional material (Additional file 3 and Additional file 4), we use a questionnaire-based prediction model for age to justify this exclusion. Using the same prediction model, we consider a second exclusion of outliers for predicted age in the additional material (Additional file 3). We report which results are significantly affected by this further exclusion.

For the first analysis, we excluded participants without matched counterparts, i.e. patients with no matched control or controls with no matched patient. The matched analysis then compared 469 patients with 624 matched controls.

**Fig. 1:**
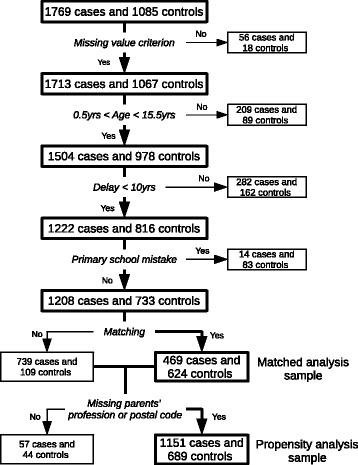
Flowchart of the samples definition. Missing value criterion is verified if at least half the questionnaire was filled. Delay refers to the time between diagnosis and questionnaire reception. Participants have made the primary school mistake if they answered that they went to primary school even though their reference age is smaller than 5.5 years. The two samples on which analyses were performed are in the bottom right corner

We also performed a propensity analysis without using the matching. We only excluded participants with no available postal code or parents’ profession as these variables were used to control for bias. This resulted in a sample of 1151 patients and 689 controls. The processes of exclusion and sample definition are summarized in Fig. 1.

### Analytical procedures

#### Matched analysis

We used methods that take matching into account and allow for variable size of the matched strata: either one patient and one control or one patient and two controls. For questions with binary responses, we performed Cochran-Mantel-Haenszel tests and for ordinal responses, we performed conditional logistic regression [38]. In both cases, we used the strata defined by the matching and the disease status as outcome. To avoid convergence problems, we excluded variables with a standard deviation smaller than 0.1.

### Propensity analysis

In this second analysis, we used stratification on the propensity score [39] to control for bias. Propensity score methods allow to control for bias by comparing participants with a similar probability of treatment (here the response to a question) given the covariates defined below.

Random Forests [40] p587-604 is a popular machine learning algorithm praised for its state-of-the-art predictive performance. Furthermore, it provides a reliable prediction on the training set called out-of-bag estimate which is not prone to overfitting. We used the randomForest package in R [41]. We trained a Random Forests regression to predict the treatment status using as predictors reference age, socio-economic status, urban/rural environment and study center. We then defined the propensity score as the out-of-bag estimate of the random forest. We then stratified our sample in 10 strata according to deciles of the propensity score and performed a Cochran-Mantel-Haenszel test (respectively a conditional logistic regression) between the question of interest if it was binary (respectively if it was ordinal) and disease status. We again excluded variables whose standard deviation was smaller than 0.1.

### Covariate description

The following covariates were used to define the propensity score for the propensity analysis:

#### Age

The reference age was written on the first page of the questionnaire as an integer number of years that corresponds to a rounding of the patient’s age at diagnostic. In both analyses, we used non-rounded patient’s age at diagnostic for both the patient and his matched controls.

#### Socio-economic status

Socio-economic status was assessed using the hand-written professions of parents. It was encoded as an ordinal variable taking value 0, 1 or 2 where 0 corresponds to blue-collar workers, 1 to intermediate professions and 2 to upper class. Among the 1840 participants of the propensity analysis, 837 were classified as 0, 725 as 1, and 278 as 2.

#### Urban/farm environment

Using the postal code of the participants obtained through the questionnaire, two variables defined at the level of the patient’s “commune” (town) of residence were used to quantify whether the participants lived in an urban or rural area. Those variables are the urban units index (as a code reflecting the size of the commune’s urban area) and the percentage of farmers in the active population. Those two variables came from anonymous public databases (French Quetelet Network (http://www.reseau-quetelet.cnrs.fr), via the Centre Maurice Halbwachs –Archives de Données Issues de la Statistique Publique (http://www.cmh.ens.fr/greco/adisp.php)) and were dated in 2007 (census closest to the date that patients started to receive the environmental questionnaire). Environment was also controlled by the recruitment center e.g. the hospital or pediatric endocrinology practice that recruited the patient: each center with more than 30 participants was coded as a distinct binary variable.

#### Correction for multiple tests

To control for multiple testing, we used the Bonferroni correction which allows to control the family-wise error rate at 5 %. For the matched analysis, as we consider that it is of better quality than the propensity analysis, we also considered the more lenient false discovery rate [42] for a level of 5 %.

We report the list of variables that passes both the FDR threshold for the matched analysis and the Bonferroni threshold for the propensity analysis. This provides better control over false positives than considering only one of the two thresholds. We also report results for variables associated with T1D in the literature.

## Results

Demographic and quality indicators of the two samples are available in Table 1.

**Table 1:** Characteristics of cases and controls in the two samples

	Matched analysis sample		Propensity analysis sample	
	Cases	Controls	Cases	Controls
Number	469	624	1151	689
Age (years)	7.5 (4.2;10.5)	7.7 (4.6;10.5)	7.6 (4.2;10.6)	7.8 (4.6;10.5)
Delay (years)	3.0 (1.0;5.2)	2.9 (1.1;5.2)	2.9 (0.9;5.6)	3.0 (1.1;5.4)
Missing data (%)	4.4 (2.7;6.8)	3.9 (2.5;6.0)	4.9 (3.1;7.5)	3.8 (2.4;5.9)

Age is the reference age. Delay is the time between the diagnosis date and the questionnaire reception. The values displayed are the median value and the first and third quartile between parentheses

### Matched analysis

For convenience, the variables have been labeled in the figures. Correspondence between labels and precise description of variables are available in Additional file 2.

**Fig. 2:**
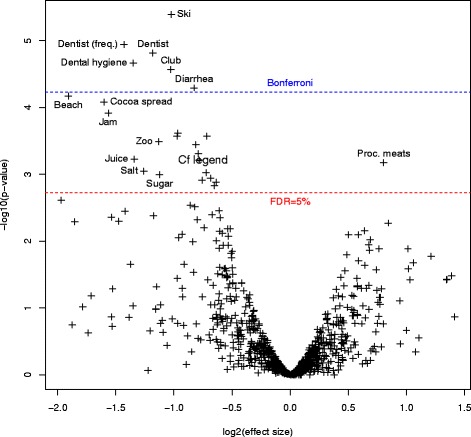
Volcano plot for the matched analysis. The x-axis shows the effect size with protective factors on the left and risk factors on the right. The y-axis indicates the significance. The higher line indicates the Bonferroni threshold while the lower line shows the more lenient threshold for 5 % of false discovery rate. The unlabeled variables above the FDR threshold are from most significant to least: week-ends with other children, taste for sugar as a baby, death of a pet from old age, vegetables from farm, home-made delicatessen, stings (mainly wasps and bees), siblings before birth, friend’s pool, plane, fresh exotic fruits, vegetables from a rural market during pregnancy

Figure 2 presents a volcano plot where both the effect size and the significance of answers to each question are displayed. We also display in blue the Bonferroni-Holm threshold for multiple testing, this means that we control the family-wise error rate at 5 % for the list of variable over the blue line. The more lenient threshold for a false discovery rate of 5 % is displayed in red. Questions that pass this threshold are labeled in the plot. Exact sample size, *p*-value, estimate and confidence interval for each variable are available as Additional file 2.

Three questions showed that cases more often had a relative with T1D and are excluded from the plots and discussion.

### Propensity analysis

**Fig. 3:**
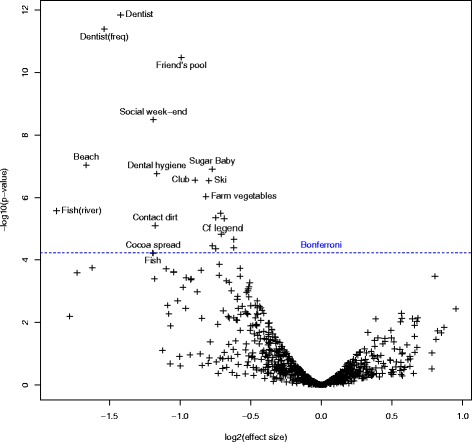
Volcano plot for the propensity analysis. The x-axis shows the effect size with protective factors on the left and risk factors on the right. The y-axis indicates the significance. The horizontal line indicates the Bonferroni threshold. The unlabeled variables above the threshold are from most significant to least: fruits from a farm or a family garden during childhood, stings, diarrhea, diarrhea during winter, contact with cats in the neighborhood, pet shop, swimming pool during pregnancy and death of a pet of old age

Results are also available in Additional file 2. They are shown in Fig. 3.

### Comparison

**Fig. 4:**
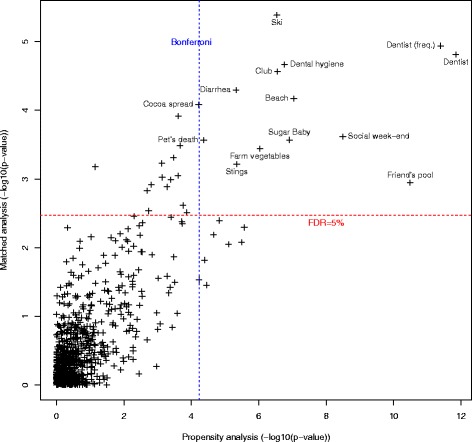
Comparison of the results of the two analysis. -log10 (*p*-value) of the two analysis plotted against each other. The most associated variables in both analysis are in the top right corner. The Bonferroni threshold for the propensity analysis is the vertical line. The false discovery rate threshold for the matched analysis is the horizontal line. A more lenient statistical control is used for the matched analysis as it is less prone to bias. All variables passing both thresholds are labeled

The result of the two analyses are summarized in Fig. 4. Table 2 provides more information on the results of the significant variables.

**Table 2:** Effect sizes for significant variables and pending risk factors

		Matched analysis			Propensity analysis		
Label	Levels	Missing	Effect size	(CI)	Missing	Effect size	(CI)
Club^b^	2	1 %/2 %	0.49	(0.35;0.68)	2 %/2 %	0.54	(0.42 0.68)
Social week-end	2	1 %/1 %	0.51	(0.36;0.73)	1 %/1 %	0.44	(0.33;0.58)
Friend’s pool	2	1 %/0 %	0.62	(0.47;0.82)	1 %/0 %	0.5	(0.41;0.62)
Ski	2	1 %/2 %	0.49	(0.36;0.67)	2 %/2 %	0.58	(0.47;0.71)
Beach^a^	4	3 %/2 %	0.27	(0.14;0.51)	3 %/2 %	0.32	(0.20;0.49)
Diarrhea	2	5 %/5 %	0.56	(0.43;0.74)	7 %/5 %	0.62	(0.51;0.76)
Cocoa spread	5	1 %/1 %	0.33	(0.19;0.57)	0 %/1 %	0.44	(0.29;0.66)
Sugar baby	2	2 %/3 %	0.61	(0.47;0.79)	3 %/2 %	0.59	(0.48;0.71)
Dental hygiene^b^	3	0 %/0 %	0.39	(0.25;0.6)	1 %/0 %	0.45	(0.33;0.61)
Dentist^a^	2	3 %/1 %	0.44	(0.3;0.64)	3 %/3 %	0.37	(0.28;0.49)
Dentist (freq.)^a^	4	2 %/3 %	0.37	(0.24;0.58)	2 %/1 %	0.34	(0.25;0.47)
Stings	2	3 %/3 %	0.58	(0.43;0.79)	3 %/3 %	0.6	(0.48;0.74)
Pet’s death	2	14 %/12 %	0.51	(0.35;0.73)	13 %/11 %	0.6	(0.47;0.76)
Farm vegetables	2	1 %/1 %	0.57	(0.42;0.77)	1 %/1 %	0.57	(0.45;0.71)
Exclusive breastfeeding	2	2 %/2 %	0.88	(0.68;1.15)	2 %/2 %	0.77	(0.63;0.94)
Respiratory infections	2	5 %/4 %	0.87	(0.68;1.12)	6 %/4 %	0.89	(0.73;1.1)

Effect sizes are odd ratios for binary variables and correspond to odd ratio between extreme responses for ordinal variables. Percentage of missing data are split between patients and controls. Factors from the literature are at the end of the table

^a^variables affected by further age-related exclusion

^b^variables affected by the further exclusion for the propensity analysis only

Social variables and markers of outdoor life are negatively associated with T1D: club attendance, playing with friends during the week-end, going to the pool at a friend’s house, winter sports and going often to the beach. Going often to the beach was sensitive to the age-related exclusion considered in the additional material (Additional file 3). Club attendance was also partially affected.

Patients had less gastroenteritis before T1D diagnosis.

Hazelnut cocoa spread consumption and sweet eating as a baby were both negatively associated with T1D.

Three variables were closely connected to dental hygiene. The variable “dental hygiene” is an ordinal variable quantifying the frequency at which the participants brush their teeth. The two variables “dentist” and “dentist (freq.)” are a binary and an ordinal variable quantifying the number of dentist visit attended by the participant. Future T1D patients attended the dentist less and brushed their teeth less as well. The association for dentist attendance was very sensitive to the further exclusion considered in the additional material (Additional file 3). Dental hygiene was also partially affected.

The patients reported having been stung less than controls. “Stings” refers to the question: Was the subject stung by an animal who left a clear spot (red spot, painful or not)? with four propositions for the responsible animal: a wasp, a bee, another insect or a fish. Mosquitoes, spiders and ticks were the subjects of separate questions. Wasp and bee stings were the most common stings.

Patients less often had the experience of having a pet die of old age.

Patients ate less vegetables coming from a farm or a family garden.

### Factors studied in the literature

We compared the results of our study with the few risk factors that have been suspected to be associated with T1D in the studies cited in the introduction.

Breastfeeding was investigated by two questions in the questionnaire: whether the subject had been breastfed at all and the duration of exclusive breastfeeding. In the matched analysis, neither questions were significant at the nominal level but in the propensity analysis, the duration of exclusive breast-feeding was found to be highly protective. Any breastfeeding was also protective with nominal significance.

Lower respiratory infections were not associated with risk of T1D in our analyses.

Vitamin D supplementation for the mother after birth was not associated with T1D in either analysis.

## Discussion

While our statistical analysis indicates that playing with friends during week-ends or going to the pool at a friend’s house and experience of winter sports were all negatively associated to childhood T1D, we have not attempted to interpret these protective associations.

We also found a negative association of gastroenteritis and T1D. Gut microbiology is an area highlighted by this observation. Sub-questions regarding gastroenteritis reveal that the negative association holds for diarrhea during winter and in the context of familial diarrhea. The results of the DAISY study suggested a more complex relationship with gastroenteritis [17].

As sugar consumption is strongly present as a nutritional caveat in the minds of parents having a child with T1D, we suspected that the negative association between “appetite for sugar as a baby” and T1D could be due to recall bias. However, with respect to a possible recall bias, sugary products such as cola drinks or chocolate show no association with T1D. This gives credibility to the found negative association for hazelnut cocoa spread. Furthermore, hazelnut cocoa spread remains significant after adjustment for appetite for sugar as a baby: in the matched sample, fitting a conditional logistic regression to both variables gives an estimate for cocoa spread of 0.36 (0.20,0.64) instead of 0.33 (0.19,0.57), meaning that the result for cocoa spread is not affected by recall bias. Hazelnut cocoa spread contains a large proportion of palm oil thus a high content of tocotrienol. In murine models, tocotrienol was shown to affect NLPR3 and NF-kB [43, 44], which may play a role in T1D pathogenesis [45, 46].

We found that items related with dental hygiene, such as frequency of teeth brushing and dentist attendance, were negatively associated with T1D although they were sensitive to a further exclusion. Again, we have not attempted to interpret this protective association in our current state of knowledge.

Wasp and bee stings also showed a significant association with T1D, but the meaning of this observation remains to be found.

Death of pet by old age was negatively associated with T1D. This was a subquestion of death of a pet which was nominally significant in both analysis. Another subquestion, death of a cat, was also associated. We offer no interpretation.

Eating vegetables from a farm or a family garden was negatively associated with T1D. The analogue question for fruits passed the Bonferroni threshold in the propensity analysis and was also nominally significant in the matched analysis. These associations might be connected to contact with dirt which was also significant in the propensity analysis and nominally significant in the matched analysis. Again, we offer no interpretation.

## Conclusion

While many exposures and events have remained out of reach of our questionnaire because they were not detectable or escaped parental memory, the novel protective associations that were found cannot be entirely false positive findings. They may open new areas of investigation for T1D environmental research and should not be dismissed more than yet biologically inexplicable SNP associations generated by GWAS. However they will only be of interest if they can be confirmed in other childhood T1D cohorts.

## Additional files

Additional file 1: The questionnaire used in the current study. (PDF 620 kb) Additional file 2: The results of matched analysis and propensity analysis including questionnaire section, question description, labels used in figures, missing data separately for cases and controls, *p*-values, effect size ad confidence interval. (XLS 355 kb) Additional file 3: Additional analysis supporting the exclusion for primary school mistake and exploring further age-related bias. (PDF 5474 kb) Additional file 4: Results of the analysis described in Additional file 3. (XLS 12 kb)
