# Supplementary material for: Association of environmental markers with childhood type 1 diabetes mellitus revealed by a long questionnaire on early life exposures and lifestyle in a case–control study
Source: BMC Public Health. 2016 Sep 29;16:1021. doi: 10.1186/s12889-016-3690-9 (PMC5041527; doi:10.1186/s12889-016-3690-9)
Supplement: Additional file 1: — The questionnaire used in the current study. (PDF 620 kb) [file 12889_2016_3690_MOESM1_ESM.pdf]

ISIS-DIAB
Cohorte Française sur les facteurs de risque génétiques et environnementaux du diabète auto-immun et de ses complications
Assistance Publique-Hôpitaux de Paris

ETUDE SUR L'ENVIRONNEMENT AVANT LA SURVENUE DU DIABÈTE CHEZ L'ENFANT

Questionnaire destiné aux patients et à leur famille

Bonjour,

Vous avez accepté de répondre à ce questionnaire concernant votre environnement, nous vous en remercions. Vos réponses seront une aide précieuse dans la recherche des facteurs environnementaux impliqués dans la survenue du diabète. Cette enquête n'a pas pour but de trouver directement la cause du diabète mais de mettre les chercheurs sur des pistes de recherche.

Ce questionnaire concerne la santé et l'environnement de l'enfant diabétique avant sa naissance et dans les années qui ont précédé le diagnostic du diabète ; quelques questions concernent la grand-mère maternelle du patient, d'autres sa mère, et son père.
Les renseignements qui seront collectés resteront strictement confidentiels et ne seront pas rendus publics. Leur exploitation sera réservée aux médecins et aux équipes qui mènent cette recherche, tous soumis au secret médical. Les données informatisées seront stockées de façon anonyme.

COMMENT RÉPONDRE AU QUESTIONNAIRE ?

Remplir ce questionnaire devrait prendre entre 1 heure 30 et 2 heures. L'enfant diabétique sera toujours désigné comme «le patient».
Le questionnaire est organisé en plusieurs sections : afin d'en faciliter l'analyse, nous avons rassemblé des questions ouvertes (professions, dates, adresses...) dans une première partie, les suivantes ne comprennent que des questions avec des cases à cocher.
Plusieurs questions concernent la mère du patient, vous aurez donc à faire appel à votre mère pour y répondre ; pour d'autres, vous aurez besoin des carnets de santé.

*Nous vous remercions d'utiliser **un stylo-bille noir ou bleu** (pas de stylo plume, de feutre, de crayon noir...) pour remplir ce questionnaire.*

- *Remplissez les espaces destinés au recueil des informations. Exemples :*
  Date de naissance du patient : |_1_|_4_| |_0_|_3_| |_1_|_9_|_9_|_9_|
  Profession du père du patient : agriculteur

- *Cochez la case correspondant à votre choix. Par défaut, ne cochez qu'une case. Exemple :*
  Le patient a-t-il été au zoo ?

  ☐1 *Une fois* ☐2 *Plusieurs fois* ☒3 *Jamais*

- *Attention, dans certains cas, vous serez amené à passer des questions qui ne vous concernent pas ; suivez les indications qui vous seront données.*

© 2008-2016, INSERM U 1169

1. **Nom et prénom du patient :** ....

1 - Questions ouvertes concernant le patient et sa famille

1.1 - Les grands-parents

2. **La grand-mère maternelle est-elle la mère biologique de la mère du patient?**

☐ 1 Oui ☐ 2 Non ⇨ *Allez à la question 3*

2.1. **Si oui, quelle est la date de naissance de la grand-mère ?**

|___|___| |___|___| |___|___|___|___|

2.2. **Quel est son pays d'origine** ....

2.3. **Quelle était l'adresse de cette grand-mère lorsqu'elle était enceinte de la mère du patient ?**

Bâtiment ....

Numéro .... Voie ....

Code postal .... Ville ....

Pays ....

2.4. **Quel était le métier de la grand-mère maternelle ?** ....

2.5. **La grand-mère a-t-elle été malade lorsqu'elle était enceinte de la mère du patient ?**

2.5.1. ☐ 1 Oui **Si oui, quelle était cette maladie ?** ....

....

☐ 2 Non

3. **Quel est le pays d'origine du grand-père maternel ?** ....

4. **Quel était son métier ?**...

5. **Quel est le pays d'origine de la grand-mère paternelle ?**...

6. **Quel était son métier ?**...

7. **Quel est le pays d'origine du grand-père paternel ?** ....

8. **Quel était son métier ?**...

9. **Un des grands-parents a-t-il eu une ou plusieurs maladies sérieuses (c'est-à-dire avec un diagnostic) pendant la petite enfance du patient ?**

9.1. ☐ 1 Oui **Si oui :** **Qui ?** **Quelle maladie ?**

....

....

....

☐ 2 Non

© 2008-2016, INSERM U 1169

1.2 - La mère du patient

10. **La mère est-elle la mère biologique du patient ?**

☐ 1 Oui ☐ 2 Non ⇨ *Allez directement à la question 11 page 7*

La mère du patient avant sa naissance

10.1. **Quelle est la date de naissance de la mère du patient ?**

|___|___| |___|___| |___|___|___|___|

10.2. **Quel était le poids de naissance de la mère du patient ?** |___|___|___|___| g

10.3. **A quel âge la mère a-t-elle eu ses premières règles ?** |___|___| ans et |___|___| mois

10.4. **Quel était son poids approximatif à 20 ans ?** |___|___|___| kg

10.5. **Quelle est sa taille adulte ?** |___| , |___|___| m

Les adresses de la mère du patient

10.6. **Quelle était l'adresse ou les principales adresses de la mère <u>pendant son enfance et son adolescence</u> en commençant par celle où elle a passé le plus de temps ?**

Bâtiment ....

Numéro .... Voie ....

Code postal .... Ville ....

Pays ....

- - - - - - - - - - - - - - -

Bâtiment ....

Numéro .... Voie ....

Code postal .... Ville ....

Pays ....

- - - - - - - - - - - - - - -

Bâtiment ....

Numéro .... Voie ....

Code postal .... Ville ....

Pays ....

10.7. **Quelle était l'adresse de la mère <u>juste avant la grossesse</u> qui a donné naissance au futur diabétique ?**

Bâtiment ....

Numéro .... Voie ....

Code postal .... Ville ....

Pays ....

© 2008-2016, INSERM U 1169

10.8. **<u>Pendant cette grossesse,</u> l'adresse de la mère était-elle la même que précédemment ?**

☐ 1 Oui ☐ 2 Non

10.8.1. **Si non, quelle était l'adresse de la mère <u>pendant cette grossesse</u> ?**

Bâtiment ....

Numéro ....Voie ....

Code postal ....Ville ....

Pays ....

10.9. **<u>Entre la naissance du patient et le diagnostic du diabète</u>, l'adresse de la mère était-elle la même que précédemment ?**

☐ 1 Oui ☐ 2 Non

10.9.1. **Si non, quelle était l'adresse de la mère entre la naissance du patient et le diagnostic du diabète ?**

Bâtiment ....

Numéro ....Voie ....

Code postal ....Ville ....

Pays ....

Les professions de la mère du patient

10.10. **Quelles ont été les professions exercées par la mère du patient avant cette grossesse?** ....

....

....

10.11. **Quelle était la profession de la mère <u>juste avant</u> cette grossesse ?** ....

....

10.12. **La mère travaillait-elle pendant cette grossesse ?**

10.12.1. ☐ 1 Oui **Si oui, cette profession était-elle différente** ?

10.12.1.1. ☐ 1 Oui **Si oui, quelle était la profession de la mère ?**

....

☐ 2 Non

☐ 2 Non ⇨ *Allez à la question 10.13 page 5*

10.12.2. **Quelle était l'adresse de son lieu de travail ?**

Bâtiment ....

Numéro ....Voie ....

Code postal ....Ville ....

Pays ....

10.12.3. **Se rendait-elle à son travail pendant cette grossesse en utilisant les transports en commun ?**

☐ 1 Oui ☐ 2 Non

© 2008-2016, INSERM U 1169

10.12.4. **A-t-elle bénéficié d'un arrêt de travail pendant cette grossesse -en dehors du congé de maternité légal ?**

10.12.4.1. ☐ 1 Oui **Si oui, à quel mois de grossesse ?** |___| mois

☐ 2 Non

10.13. **Entre la naissance du patient et la date du diagnostic du diabète, la profession de la mère était-elle différente de celle exercée précédemment ?**

10.13.1. ☐ 1 Oui **Si oui, quelle était cette profession ?**

....

☐ 2 Non

La santé de la mère du patient

Pour les questions qui suivent, aidez-vous du ***carnet de santé***

**La mère a-t-elle eu au cours de sa propre enfance :**	Oui	Non	Ne sait pas
10.14. **La rougeole?**	☐ 1	☐ 2	☐ 3

10.14.1. **Si oui, à quelle date** (si vous n'avez pas l'information exacte, merci d'indiquer l'année) **?**

Date : |___|___| |___|___| |___|___|___|___|

	Oui	Non	Ne sait pas
10.15. **Les oreillons ?**	☐ 1	☐ 2	☐ 3

10.15.1. **Si oui, à quelle date** (si vous n'avez pas l'information exacte, merci d'indiquer l'année) **?**

Date : |___|___| |___|___| |___|___|___|___|

	Oui	Non	Ne sait pas
10.16. **La varicelle ?**	☐ 1	☐ 2	☐ 3

10.16.1. **Si oui, à quelle date** (si vous n'avez pas l'information exacte, merci d'indiquer l'année) **?**

Date : |___|___| |___|___| |___|___|___|___|

	Oui	Non	Ne sait pas
10.17. **La mère a-t-elle été vaccinée contre la rougeole?**	☐ 1	☐ 2	☐ 3

10.17.1. **Si oui, à quelle date** (si vous n'avez pas l'information exacte, merci d'indiquer l'année) **?**

Date : |___|___| |___|___| |___|___|___|___|

10.18. **Avant la naissance du patient, la mère avait-elle eu d'autres enfants ?**

10.18.1. ☐ 1 Oui **Si oui, combien ?** |___|___| enfants

10.18.2. **Quelle est leur date de naissance et leur sexe ?**

Date : |___|___| |___|___| |___|___|___|___|1 Sexe : |___|2 F ou M

Date : |___|___| |___|___| |___|___|___|___|3 Sexe : |___|4 F ou M

Date : |___|___| |___|___| |___|___|___|___|5 Sexe : |___|6 F ou M

Si vous souhaitez mentionner d'autres enfants, merci d'utiliser une feuille vierge à la fin de ce questionnaire en indiquant bien le numéro de la question : 10.18.2

☐ 2 Non

10.19. **Auparavant, la mère avait-elle fait des fausses-couches ?**

10.19.1. ☐ 1 Oui **Si oui, combien ?** |___|

☐ 2 Non

10.20. **Auparavant, la mère avait-elle procédé à des interruptions volontaires de grossesses ?**

10.20.1. ☐ 1 Oui **Si oui, combien ?** |___|

☐ 2 Non

© 2008-2016, INSERM U 1169

Attention, la grossesse dont il s'agit est celle qui s'est achevée par la naissance du patient diabétique

10.21. **La mère du patient avait-elle une maladie chronique avant cette grossesse ?**

10.21.1. ☐ 1 Oui **Si oui, laquelle ?** ....

....

☐ 2 Non

10.22. **Prenait-elle des médicaments avant cette grossesse ?**

10.22.1. ☐ 1 Oui **Si oui, lesquels ?** ....

....

....

☐ 2 Non

**La mère a-t-elle eu pendant sa grossesse :**	Oui	Non	Ne sait pas
10.23. **La rougeole?**	☐ 1	☐ 2	☐ 3

10.23.1. **Si oui, à quelle date** (si vous n'avez pas l'information exacte, merci d'indiquer le mois) **?**

Date : |___|___| |___|___| |___|___|___|___|

	Oui	Non	Ne sait pas
10.24. **Un contact avec un rougeoleux ?**	☐ 1	☐ 2	☐ 3

10.24.1. **Si oui, à quelle date** (si vous n'avez pas l'information exacte, merci d'indiquer le mois) **?**

Date : |___|___| |___|___| |___|___|___|___|

	Oui	Non	Ne sait pas
10.25. **Les oreillons ?**	☐ 1	☐ 2	☐ 3

10.25.1. **Si oui, à quelle date** (si vous n'avez pas l'information exacte, merci d'indiquer le mois) **?**

Date : |___|___| |___|___| |___|___|___|___|

	Oui	Non	Ne sait pas
10.26. **La varicelle ?**	☐ 1	☐ 2	☐ 3

10.26.1. **Si oui, à quelle date** (si vous n'avez pas l'information exacte, merci d'indiquer le mois) **?**

Date : |___|___| |___|___| |___|___|___|___|

	Oui	Non	Ne sait pas
10.27. **La grippe ?**	☐ 1	☐ 2	☐ 3

10.27.1. **Si oui, à quelle date** (si vous n'avez pas l'information exacte, merci d'indiquer le mois) **?**

Date : |___|___| |___|___| |___|___|___|___|

10.28. **Quelle est l'adresse du lieu de naissance du futur diabétique (maternité, hôpital...) ?**

Bâtiment ....

Numéro ....Voie ....

Code postal ....Ville ....

Pays ....

© 2008-2016, INSERM U 1169

10.29. **La mère a-t-elle subi une intervention chirurgicale pendant l'enfance du futur diabétique ?**

10.29.1. ☐ 1 Oui **Si oui, combien ?** |___|

10.29.2. **Laquelle ou lesquelles ?** ....

....

....

☐ 2 Non

1.3 - Le père du patient

11. **Quelle est la date de naissance du père du patient ?** |___|___| |___|___| |___|___|___|___|

12. **Quelle était la profession exercée par le père avant cette grossesse ?** ....

....

13. **La profession du père <u>pendant la grossesse</u> était-elle différente ?**

13.1. ☐ 1 Oui **Si oui, quelle profession exerçait le père durant cette période ?**..............................

....

☐ 2 Non

14. **La profession du père <u>entre la naissance du patient et le diagnostic du diabète</u> était-elle différente ?**

14.1. ☐ 1 Oui **Si oui, quelle profession exerçait le père durant cette période ?**..............................

....

☐ 2 Non

15. **Le père a-t-il subi une intervention chirurgicale pendant l'enfance du futur diabétique ?**

15.1. ☐ 1 Oui **Si oui, combien ?** |___|

15.2. **Laquelle ou lesquelles ?**..

....

☐ 2 Non

16. **Le père du patient avait-il une maladie chronique ?**

16.1. ☐ 1 Oui **Si oui, laquelle ?** ....

....

☐ 2 Non

**Le père a-t-il été vacciné contre une des maladies suivantes :**	Oui	Non
17. **La rougeole** ?	☐ 1	☐ 2
18. **Les oreillons** ?	☐ 1	☐ 2

1.4 - Le patient entre sa naissance et la découverte du diabète

*Pour les questions qui suivent, aidez-vous du **carnet de santé** !*

19. **Quelle est la date de naissance du patient ?** |___|___| |___|___| |___|___|___|___|

20. **Si vous vous le rappelez, quel est le terme précis de l'accouchement ?** |___|___| semaines

21. **Quel était son poids à la naissance ?** |___|___|___|___| g

© 2008-2016, INSERM U 1169

22. **Quelle était sa taille à la naissance ?** |___|___| cm

Quel était son poids à :

23. **Environ 1 mois (carnet de santé) ?** |___|___|___|___| g

24. **Environ 3 mois (carnet de santé) ?** |___|,|___|___| kg

25. **Environ 6 mois (carnet de santé) ?** |___|,|___|___| kg

26. **Environ 1 an (carnet de santé) ?** |___|___|,|___|___| kg

27. **Environ 18 mois (carnet de santé) ?** |___|___|,|___|___| kg

28. **Environ 2 ans (carnet de santé) ?** |___|___|,|___|___| kg

29. **A quel âge a-t-il marché ?** |___|___| mois

La santé du patient

30. **Le patient a-t-il eu une maladie particulière diagnostiquée pendant sa première année?**

30.1. ☐ 1 Oui **Si oui, laquelle ?**...

☐ 2 Non

<u>**Avant le diagnostic du diabète**</u>**, le patient a-t-il eu :** Oui Non Ne sait pas

31. **La rougeole?** .... ☐ 1 ☐ 2 ☐ 3

31.1. **Si oui, à quelle date** (si vous n'avez pas l'information exacte, merci d'indiquer l'année) **?**

Date : |___|___| |___|___| |___|___|___|___|

32. **Un contact avec un rougeoleux ?** .... ☐ 1 ☐ 2 ☐ 3

32.1. **Si oui, à quelle date** (si vous n'avez pas l'information exacte, merci d'indiquer l'année) **?**

Date : |___|___| |___|___| |___|___|___|___|

33. **Les oreillons ?** .... ☐ 1 ☐ 2 ☐ 3

33.1. **Si oui, à quelle date** (si vous n'avez pas l'information exacte, merci d'indiquer l'année) **?**

Date : |___|___| |___|___| |___|___|___|___|

34. **La varicelle ?** .... ☐ 1 ☐ 2 ☐ 3

34.1. **Si oui, à quelle date** (si vous n'avez pas l'information exacte, merci d'indiquer l'année) **?**

Date : |___|___| |___|___| |___|___|___|___|

34.2. **a-t-il pris des antibiotiques ?** .... ☐ 1 ☐ 2 ☐ 3

35. **La grippe ?** .... ☐ 1 ☐ 2 ☐ 3

35.1. **Si oui, à quelle date** (si vous n'avez pas l'information exacte, merci d'indiquer l'année) **?**

Date : |___|___| |___|___| |___|___|___|___|

36. **Le patient a-t-il été vacciné contre la rougeole ?** .... ☐ 1 ☐ 2 ☐ 3

36.1. **Si oui, à quelle date** (si vous n'avez pas l'information exacte, merci d'indiquer l'année) **?**

Date : |___|___| |___|___| |___|___|___|___|

37. **Le patient a-t-il eu son rappel de vaccination contre la rougeole ?** .... ☐ 1 ☐ 2 ☐ 3

37.1. **Si oui, à quelle date** (si vous n'avez pas l'information exacte, merci d'indiquer l'année) **?**

Date : |___|___| |___|___| |___|___|___|___|

© 2008-2016, INSERM U 1169

38. **Entre la naissance et la découverte de son diabète, le patient avait-il subi une intervention chirurgicale ?**

☐ 1 Oui ☐ 2 Non ⇨ *Allez directement à la question 39*

38.1. **Si oui, la ou lesquelles ?**

Cause	Age	Anesthésie générale	
....	|___|___|1 ans	☐ 2 Oui	☐ 3 Non
....	|___|___|4 ans	☐ 5 Oui	☐ 6 Non
....	|___|___|7 ans	☐ 8 Oui	☐ 9 Non

Si vous souhaitez ajouter des informations, merci d'utiliser une feuille vierge à la fin de ce questionnaire en indiquant bien le numéro de la question : 38.1

39. **Entre la naissance et la découverte de son diabète, le patient avait-il été hospitalisé?**

☐ 1 Oui ☐ 2 Non ⇨ *Allez directement à la question 40*

39.1. **Si oui, pour quelle raison ?** ....

dans quel hôpital ou clinique ?

Hôpital ou clinique...

Numéro ....Voie ....

Code postal ....Ville ....

Pays ....

pour quelle raison ? ....

dans quel hôpital ou clinique ?

Hôpital ou clinique...

Numéro ....Voie ....

Code postal ....Ville ....

Pays ....

Si vous souhaitez ajouter des informations, merci d'utiliser une feuille vierge à la fin de ce questionnaire en indiquant bien le numéro de la question : 39.1

40. **A quelle date le diabète a-t-il été diagnostiqué ?**

|___|___| |___|___| |___|___|___|___|

Les adresses du patient

41. **Combien de fois le patient a-t-il déménagé entre sa naissance et le diagnostic du diabète?**

|___|___| fois

42. **A quelle adresse vivait le patient entre sa naissance et la découverte du diabète, en cas de déménagements mentionnez en premier l'adresse où il a passé le plus de temps ?**

Bâtiment...

Numéro ....Voie ....

Code postal ....Ville ....

Pays ....

- - - - - - - - - - - - - - -

© 2008-2016, INSERM U 1169

Bâtiment ....

Numéro .... Voie ....

Code postal .... Ville ....

Pays ....

1.5 - La vie de la famille

Les autres enfants

43. **Combien de frères et de sœurs vivaient au domicile familial entre la naissance et le diagnostic du diabète (ne pas compter le patient) ?** |___|___| enfants

44. **Des frères ou des sœurs sont-ils nés entre la naissance du patient et le diagnostic de son diabète ?**

44.1. ☐ 1 Oui **Si oui, quelle est leur date de naissance et leur sexe ?**

Date : |___|___| |___|___| |___|___|___|___|1 Sexe : |___|2 F ou M

Date : |___|___| |___|___| |___|___|___|___|3 Sexe : |___|4 F ou M

Date : |___|___| |___|___| |___|___|___|___|5 Sexe : |___|6 F ou M

Si vous souhaitez ajouter des enfants, merci d'utiliser une feuille vierge à la fin de ce questionnaire en indiquant bien le numéro de la question : 44.1

☐ 2 Non

*Pour les questions qui suivent, aidez-vous du **carnet de santé** !*

45. **Un frère ou une sœur a-t-il subi une ou plusieurs autres maladies sérieuses (c'est-à-dire avec un diagnostic) pendant la petite enfance du futur diabétique ?**

☐ 1 Oui ☐ 2 Non

Si oui :

45.1.

**Quel enfant ?**	**Quelle maladie ?**
....	....
....	....
....	....

Si vous souhaitez ajouter des enfants, merci d'utiliser une feuille vierge à la fin de ce questionnaire en indiquant bien le numéro de la question : 45.1

46. **Un frère ou une sœur a-t-il subi une intervention chirurgicale pendant cette période ?**

☐ 1 Oui ☐ 2 Non

Si oui :

46.1.

**Quel enfant ?**	**Quelle intervention ?**
....	....
....	....
....	....

Si vous souhaitez ajouter des informations, merci d'utiliser une feuille vierge à la fin de ce questionnaire en indiquant bien le numéro de la question : 46.1

© 2008-2016, INSERM U 1169

<u>Avant le diagnostic du diabète</u>, au moins un des frères et sœurs enfant a-t-il eu :

(Si plusieurs de vos enfants ont eu une de ces maladies, indiquez la date la plus proche de la naissance du patient)

	Oui	Non	Ne sait pas
47. **La rougeole?**	☐ 1	☐ 2	☐ 3
48. **Un contact avec un rougeoleux ?**	☐ 1	☐ 2	☐ 3
49. **Les oreillons ?**	☐ 1	☐ 2	☐ 3
50. **La varicelle ?**	☐ 1	☐ 2	☐ 3
51. **La grippe ?**	☐ 1	☐ 2	☐ 3
52. **La vaccination contre la rougeole ?**	☐ 1	☐ 2	☐ 3

47. **La rougeole?** .... ☐ 1 ☐ 2 ☐ 3

47.1. **Si oui, à quelle date** (si vous n'avez pas l'information exacte, merci d'indiquer l'année) **?**

Date : |___|___| |___|___| |___|___|___|___|

48. **Un contact avec un rougeoleux ?** .... ☐ 1 ☐ 2 ☐ 3

48.1. **Si oui, à quelle date** (si vous n'avez pas l'information exacte, merci d'indiquer l'année) **?**

Date : |___|___| |___|___| |___|___|___|___|

49. **Les oreillons ?** .... ☐ 1 ☐ 2 ☐ 3

49.1. **Si oui, à quelle date** (si vous n'avez pas l'information exacte, merci d'indiquer l'année) **?**

Date : |___|___| |___|___| |___|___|___|___|

50. **La varicelle ?** .... ☐ 1 ☐ 2 ☐ 3

50.1. **Si oui, à quelle date** (si vous n'avez pas l'information exacte, merci d'indiquer l'année) **?**

Date : |___|___| |___|___| |___|___|___|___|

51. **La grippe ?** .... ☐ 1 ☐ 2 ☐ 3

51.1. **Si oui, à quelle date** (si vous n'avez pas l'information exacte, merci d'indiquer l'année) **?**

Date : |___|___| |___|___| |___|___|___|___|

52. **La vaccination contre la rougeole ?** .... ☐ 1 ☐ 2 ☐ 3

52.1. **Si oui, à quelle date** (si vous n'avez pas l'information exacte, merci d'indiquer l'année) **?**

Date : |___|___| |___|___| |___|___|___|___|

La structure de la famille

53. **Les parents ont-ils divorcé ou se sont-ils séparés entre la naissance du patient et le diagnostic de son diabète ?**

☐ 1 Oui ☐ 2 Non ⇨ *Allez à la question **53.6 page 12***

53.1. **Si oui, les parents étaient-ils séparés avec la garde alternée du patient ?**

☐ 1 Oui ☐ 2 Non

53.1.1. **Si oui, le patient vivait-il :**

☐ 1 Uniquement chez sa mère

☐ 2 Uniquement chez son père

☐ 3 Autant chez sa mère que chez son père

☐ 4 Plus fréquemment chez sa mère

☐ 5 Plus fréquemment chez son père

53.2. **Quelle était l'adresse de la mère ?**

Bâtiment ....

Numéro .... Voie ....

Code postal .... Ville ....

Pays ....

© 2008-2016, INSERM U 1169

53.3. **Combien de frères et de sœurs vivaient au domicile de la mère durant cette période (ne pas compter le patient) ?** |___|___| enfants

53.4. **Quelle était l'adresse du père ?**

Bâtiment ....

Numéro .... Voie ....

Code postal .... Ville ....

Pays ....

53.5. **Combien de frères et de sœurs vivaient au domicile du père durant cette période (ne pas compter le patient) ?** |___|___| enfants

➪ *Allez à la question 54*

53.6. **Quelle était l'adresse des parents ?**

Bâtiment ....

Numéro .... Voie ....

Code postal .... Ville ....

Pays ....

Les proches du patient

54. **Le patient était-il en contact avec un ou plusieurs adultes (autres que les grands-parents) ayant une maladie sérieuse (c'est-à-dire avec un diagnostic) pendant sa petite enfance ?**

54.1. ☐ 1 Oui **Si oui, combien ?** |___|

54.2. **Si oui : Quelles personnes ? Quelle maladie ?**

Quelles personnes ?	Quelle maladie ?
....	....
....	....
....	....
....	....

Si vous souhaitez ajouter des informations, merci d'utiliser une feuille vierge à la fin de ce questionnaire en indiquant bien le numéro de la question : 54.2

☐ 2 Non

55. **Un ou plusieurs membres de la famille ou proches du patient sont-ils décédés entre la naissance et le début du diabète ?**

55.1. ☐ 1 Oui **Si oui, quelle est la cause de ce ou ces décès ?**

....

....

....

☐ 2 Non

© 2008-2016, INSERM U 1169

2 - AVANT LA NAISSANCE DU FUTUR DIABÉTIQUE

2.1 - La famille du patient avant la grossesse

Les grands-parents maternels, oncles ou tantes ont-ils exercé l'une des professions suivantes :

		Oui	Non
56.	**Agriculteur ?**	☐ 1	☐ 2
57.	**Vétérinaire ?**	☐ 1	☐ 2
58.	**Médecin ?**	☐ 1	☐ 2
59.	**Infirmièr(e) ?**	☐ 1	☐ 2
60.	**Coiffeur(se) ?**	☐ 1	☐ 2

61. **Le père du patient portait-il des lunettes ou/et des lentilles de correction de vue?**

61.1. ☐ 1 Oui **Si oui, quelle était son acuité visuelle corrigée ?**

☐ 1 10/10 ☐ 2 9/10 ☐ 3 8/10 ☐ 4 7/10 ☐ 5 moins de 6/10

☐ 2 Non

62. **Le père du patient était-il myope ?**

62.1. ☐ 1 Oui **Si oui, connaissez-vous la puissance de sa correction ?**

☐ 1 Oui **Si oui,**

		De - 1 à - 3 dioptries	De - 3 à - 6 dioptries	Plus forte que - 6 dioptries
62.1.1.	Œil droit	☐ 1	☐ 2	☐ 3
62.1.2.	Œil gauche	☐ 1	☐ 2	☐ 3

☐ 2 Non

☐ 2 Non

2.2 - La vie de la mère du patient diabétique avant la grossesse

Les questions qui suivent concernent la mère biologique du patient diabétique
Si sa mère n'est pas sa mère biologique alors, allez directement à la question 199 page 23

63. **La mère du patient est-elle née :**

☐ 1 A terme ☐ 2 Avant terme

64. **La mère dans l'enfance était-elle ?**

☐ 1 Très mince-maigre
☐ 2 Mince
☐ 3 Normale
☐ 4 Un peu enveloppée, "ronde"
☐ 5 En net surpoids

65. **La mère à l'adolescence était-elle ?**

☐ 1 Très mince-maigre
☐ 2 Mince
☐ 3 Normale
☐ 4 Un peu enveloppée, "ronde"
☐ 5 En net surpoids

© 2008-2016, INSERM U 1169

**La mère a-t-elle :**	Oui	Non
66. **Suivi un régime pour diminuer son poids pendant son enfance ?**	☐ 1	☐ 2
67. **Suivi un régime amaigrissant pendant son adolescence ?**	☐ 1	☐ 2
68. **Suivi un régime amaigrissant pendant sa vie de jeune adulte ?**	☐ 1	☐ 2
69. **La mère a-t-elle été végétarienne pendant une de ces périodes ?**	☐ 1	☐ 2

La santé de la mère avant qu'elle ne soit enceinte du futur diabétique

70. **La mère du patient portait-elle avant la grossesse des lunettes ou/et des lentilles de correction de vue?**

70.1. ☐ 1 Oui **Si oui, quelle était son acuité visuelle corrigée ?**

☐ 1 10/10 ☐ 2 9/10 ☐ 3 8/10 ☐ 4 7/10 ☐ 5 moins de 6/10

☐ 2 Non

71. **La mère du patient était-elle myope ?**

71.1. ☐ 1 Oui **Si oui, connaissez-vous la puissance de sa correction ?**

☐ 1 Oui **Si oui,**	De - 1 à - 3 dioptries	De - 3 à - 6 dioptries	Plus forte que - 6 dioptries
71.1.1. Œil droit	☐ 1	☐ 2	☐ 3
71.1.2. Œil gauche	☐ 1	☐ 2	☐ 3

☐ 2 Non

☐ 2 Non

**La mère du patient avait-elle avant la grossesse :**	Oui	Non
72. **Une obésité ?**	☐ 1	☐ 2
73. **Un surpoids ?**	☐ 1	☐ 2
74. **De l'asthme ?**	☐ 1	☐ 2

75. **Avant la grossesse la mère avait-elle un diabète ?**

75.1. ☐ 1 Oui **Si oui, à quel âge a été diagnostiqué ce diabète ?** |___|___| ans

75.2. **était-il traité à l'insuline ?** ☐ 1 Oui ☐ 2 Non

☐ 2 Non

76. **Avant la grossesse la mère avait-elle une hypothyroïdie (thyroïdite de Hashimoto) ?**

☐ 1 Oui ☐ 2 Non

77. **Avant la grossesse la mère avait-elle une hyperthyroïdie (maladie de Basedow) ?**

☐ 1 Oui ☐ 2 Non

**La mère avait-elle eu une des maladie suivantes avant la grossesse :**	Oui	Non
78. **Un syndrome pied-main-bouche (vésicules sur les paumes, les plantes de pieds et dans la bouche) ?**	☐ 1	☐ 2
79. **Une hépatite ?**	☐ 1	☐ 2
80. **Une méningite virale ?**	☐ 1	☐ 2

81. **Avait-elle eu recours à un moyen contraceptif avant cette grossesse ?**

81.1. ☐ 1 Oui **Si oui, s'agissait-il ?**

☐ 1 d'une pilule oestro-progestative

☐ 2 d'un stérilet

☐ 3 autre

☐ 2 Non

© 2008-2016, INSERM U 1169

2.3 - La vie de la mère pendant la grossesse et à la naissance du patient diabétique

Attention, la grossesse dont il s'agit est celle qui s'est achevée par la naissance du patient diabétique

L'alimentation

82. **Pendant cette grossesse la mère consommait-elle de la viande (plusieurs réponses possibles)?**

- ☐ 1 Crue (tartare, carpaccio...)
- ☐ 2 Bleue
- ☐ 3 Saignante
- ☐ 4 A point
- ☐ 5 Très cuite
- ☐ 6 La mère ne consommait pas de viande pendant cette période

**Pendant cette grossesse la mère consommait-elle :**	Oui	Non
83. **Des légumes cultivés dans le potager familial ?**	☐ 1	☐ 2
84. **Des légumes d'un marché rural ?**	☐ 1	☐ 2
85. **De la charcuterie artisanale ou familiale ?**	☐ 1	☐ 2
86. **Des conserves familiales ?**	☐ 1	☐ 2
87. **Du poisson cuit ?**	☐ 1	☐ 2
88. **Du poisson fumé ?**	☐ 1	☐ 2
89. **Du poisson cru ?**	☐ 1	☐ 2
90. **Des coquillages crus (huîtres, palourdes...) ?**	☐ 1	☐ 2
91. **Des crustacés ?**	☐ 1	☐ 2
92. **Des fromages au lait cru ?**	☐ 1	☐ 2
93. **Des pâtisseries à la crème ?**	☐ 1	☐ 2

94. **Pendant cette grossesse la mère mangeait-elle des gâteaux et des sucreries ?**

- ☐ 1 Plusieurs fois par jour
- ☐ 2 Au moins une fois par jour
- ☐ 3 Au mois une fois par semaine
- ☐ 4 Au moins une fois par mois
- ☐ 5 Plus rarement
- ☐ 6 Jamais

95. **Pendant cette grossesse suivait-elle un régime ?**

95.1. ☐ 1 Oui **Si oui, ce régime avait-il pour but de limiter la prise de poids durant la grossesse ?**
☐ 1 Oui ☐ 2 Non

95.2. ☐ 2 Non **Si non, même sans régime véritable, la mère avait-elle essayé de ne pas prendre plus de 9-10 kg pendant la grossesse ?** ☐ 1 Oui ☐ 2 Non

96. **La mère pensait-elle que prendre trop de poids est mauvais pour la santé (aussi bien la sienne que celle du bébé) ?** ☐ 1 Oui ☐ 2 Non

© 2008-2016, INSERM U 1169

97. **Le médecin qui suivait la grossesse avait-il encouragé la mère à ne pas prendre trop de poids ?**
☐ 1 Oui ☐ 2 Non

98. **Finalement la prise de poids pendant la grossesse était :**
☐ 1 Inférieure à 10kg
☐ 2 Entre 10 et 15 kg
☐ 3 Entre 15 et 20 kg
☐ 4 Plus de 20 kg

**La mère buvait-elle :**	Oui	Non
99. **Du café ?**	☐ 1	☐ 2
100. **De l'eau du robinet ?**	☐ 1	☐ 2
101. **De l'eau minérale ?**	☐ 1	☐ 2

102. **La mère a-t-elle bu de l'alcool pendant la grossesse ?**
102.1. ☐ 1 Oui **Si oui :**
☐ 1 Au moins une fois par jour
☐ 2 Au moins une fois par semaine
☐ 3 Au moins une fois par mois
☐ 4 Moins souvent
☐ 2 Non

L'hygiène

**Durant cette grossesse, la mère :**	Oui	Non
103. **Se lavait-elle les mains avant chaque repas ?**	☐ 1	☐ 2
104. **Se lavait-elle les mains après avoir été aux toilettes ?**	☐ 1	☐ 2
105. **Utilisait-elle une crème anti-vergetures ?**	☐ 1	☐ 2

106. **La mère a-t-elle eu une épilation pendant la grossesse (plusieurs réponses possibles) ?**
106.1. ☐ 1 Oui **Si oui, comment ?**
☐ 1 Crème dépilatoire
☐ 2 Rasage
☐ 3 Epilation à la cire
☐ 4 Epilateur électrique
☐ 5 Autres
☐ 2 Non

107. **La mère s'est-elle fait tatouer pendant cette grossesse ?** ☐ 1 Oui ☐ 2 Non

108. **La mère a-t-elle fumé des cigarettes pendant cette grossesse ?**
108.1. ☐ 1 Oui **Si oui, comment ?**
☐ 1 Moins de 15 cigarettes par jour
☐ 2 Plus de 15 cigarettes par jour
☐ 2 Non

109. **La mère faisait-elle le ménage à la maison ?** ☐ 1 Oui ☐ 2 Non

© 2008-2016, INSERM U 1169

Les loisirs

Durant la grossesse, la mère : Oui / Non

		Oui	Non
110.	**Faisait-elle collage, encadrement ou peinture ?**	☐ 1	☐ 2
111.	**A-t-elle été au moins une fois à la piscine ?**	☐ 1	☐ 2
112.	**A-t-elle fait des promenades, des randonnées dans la campagne ?**	☐ 1	☐ 2
113.	**A-t-elle fait des séjours en altitude ?**	☐ 1	☐ 2
114.	**A-t-elle effectué des voyages en avion ?**	☐ 1	☐ 2

115. **Durant la grossesse, la mère a-t-elle effectué des voyages dans un pays non européen ?**

☐ 1 Oui ☐ 2 Non ⇨ *Allez à la question 116*

Si oui, dans quelle région :

		Oui	Non
115.1.	**Afrique du nord ?**	☐ 1	☐ 2
115.2.	**Reste de l'Afrique ?**	☐ 1	☐ 2
115.3.	**Amérique du nord ?**	☐ 1	☐ 2
115.4.	**Amérique centrale ou Amérique du sud ?**	☐ 1	☐ 2
115.5.	**Asie ?**	☐ 1	☐ 2
115.6.	**Océanie ?**	☐ 1	☐ 2

Durant cette grossesse, la mère :

		Oui	Non
116.	**Avait-elle un potager familial ?**	☐ 1	☐ 2
117.	**Faisait-elle du jardinage (jardin, terrasse ou balcon) ?**	☐ 1	☐ 2
118.	**Faisait-elle des travaux de bricolage dans la maison ?**	☐ 1	☐ 2

Les animaux familiers

119. **Durant cette grossesse, la mère a-t-elle été en contact direct régulier avec un animal ?**

☐ 1 Oui ⇨ *Allez à la question 119.1*

☐ 2 Non ⇨ *Allez à la question 121 page 18*

La mère a-t-elle été en contact avec :

		Oui	Non
119.1.	**Un chien ?**	☐ 1	☐ 2
119.2.	**Un rongeur (hamster, chinchilla, mulots, souris, rats...) ?**	☐ 1	☐ 2
119.3.	**Une perruche, un mainate, un perroquet ?**	☐ 1	☐ 2
119.4.	**Des pigeons ?**	☐ 1	☐ 2

119.5. **La mère a-t-elle été en contact régulier avec un chat ?**

☐ 1 Oui ⇨ *Allez à la question 119.5.1*

☐ 2 Non ⇨ *Allez à la question 119.6 page 18*

119.5.1. **La mère changeait-elle la litière du chat elle-même ?** ☐ 2 Oui ☐ 2 Non

119.5.2. **Ce chat était-il nourri avec (plusieurs réponses possibles) :**

☐ 1 De la viande crue ?

☐ 2 Des croquettes ?

☐ 3 De la nourriture en boîte ?

© 2008-2016, INSERM U 1169

119.6. **Un des animaux familiers (chat, chien, rongeur...) est-il mort pendant la grossesse ?**
☐ 1 Oui ☐ 2 Non

La santé de la mère lorsqu'elle était enceinte du futur diabétique

120. **La mère a-t-elle eu recours à une technique de procréation assistée pour obtenir cette grossesse ?**
☐ 1 Oui ☐ 2 Non

121. **Où la mère était-elle suivie pour sa grossesse (plusieurs réponses possibles) ?**
☐ 1 En cabinet ☐ 2 A l'hôpital ou en clinique

122. **La mère a-t-elle eu une prise de sang pendant cette grossesse ?**
122.1. ☐ 1 Oui **Si oui, en maternité ?** ☐ 1 Oui ☐ 2 Non
122.2. **dans un laboratoire ?** ☐ 1 Oui ☐ 2 Non
☐ 2 Non

123. **La mère a-t-elle eu une amniocentèse ?** ☐ 1 Oui ☐ 2 Non

124. **La mère prenait-elle des antivomitifs ou antinauséeux en début de grossesse ?** ☐ 1 Oui ☐ 2 Non

125. **La mère a-t-elle été hospitalisée pour des vomissements en début de grossesse ?** ☐ 1 Oui ☐ 2 Non

126. **Au cours de cette grossesse, la mère a-t-elle eu une mycose génitale ?**
☐ 1 Oui ☐ 2 Non ⇨ *Allez à la question 127*
126.1. **Si oui, a-t-elle reçu un traitement local ?**
126.1.1. ☐ 1 Oui **Si oui :** ☐ 1 Crème ☐ 2 Ovule
☐ 2 Non

**Au cours de cette grossesse, la mère a-t-elle eu :**	Oui	Non
127. **Des saignements génitaux ?**	☐ 1	☐ 2
128. **Une infection urinaire ?**	☐ 1	☐ 2
129. **Une fièvre inexpliquée ?**	☐ 1	☐ 2

Sans toutefois vous livrer à une enquête trop poussée,
pouvez-vous porter une attention particulière aux questions suivantes ?

**Au cours de cette grossesse, la mère a-t-elle eu :**	Oui	Non
130. **Un syndrome pied-main-bouche (vésicules sur les paumes, les plantes de pieds et dans la bouche) ?**	☐ 1	☐ 2
131. **Une infection respiratoire (bronchite, pneumonie) ou une bronchiolite ?**	☐ 1	☐ 2
132. **Une infection de la sphère ORL : otite, sinusite, rhume, angine, laryngite ?**	☐ 1	☐ 2
133. **Une diarrhée aiguë avec des vomissements ?**	☐ 1	☐ 2
134. **Une hépatite ?**	☐ 1	☐ 2
135. **Des épisodes fébriles répétés ?**	☐ 1	☐ 2
136. **Une méningite virale ?**	☐ 1	☐ 2

....

© 2008-2016, INSERM U 1169

137. **Au cours de cette grossesse, la mère a-t-elle eu une autre maladie infectieuse ?**

137.1. ☐ 1 Oui **Si oui, en quelle saison ?**

☐ 1 Printemps
☐ 2 Eté
☐ 3 Automne
☐ 4 Hiver

☐ 2 Non

138. **Au cours de sa grossesse, la mère a-t-elle eu une allergie cutanée ?**

138.1. ☐ 1 Oui
☐ 2 Non

139. **Au cours de sa grossesse, la mère a-t-elle eu de l'hypertension artérielle ?**

139.1. ☐ 1 Oui **Si oui, la mère prenait-elle des médicaments antihypertenseurs ?**

☐ 1 Oui ☐ 2 Non

☐ 2 Non

140. **Au cours de sa grossesse, la mère a-t-elle eu une autre maladie ?**

☐ 1 Oui ☐ 2 Non

141. **Au cours de sa grossesse, la mère a-t-elle eu des soins dentaires ?**

141.1. ☐ 1 Oui **Si oui, s'est-elle fait enlever une dent pendant la grossesse ?**

☐ 1 Oui ☐ 2 Non

☐ 2 Non

**Au cours de cette grossesse la mère :**	Oui	Non
142. **A-t-elle fait une chute avec blessure ?**	☐ 1	☐ 2
143. **A-t-elle été hospitalisée ?**	☐ 1	☐ 2
144. **A-t-elle eu une intervention chirurgicale ?**	☐ 1	☐ 2
145. **A-t-elle subi une anesthésie générale ?**	☐ 1	☐ 2
146. **Etait-elle immunisée contre la rubéole ?**	☐ 1	☐ 2
147. **Etait-elle immunisée contre la toxoplasmose ?**	☐ 1	☐ 2

148. **La mère était-elle diabétique avant la grossesse ?**

148.1. ☐ 1 Oui **Dans ce cas:**

☐ 1 Etait-elle traitée à l'insuline pendant la grossesse ?
☐ 2 Suivait-elle seulement un régime diabétique pendant la grossesse ?

☐ 2 Non

**Au cours de cette grossesse la mère :**	Oui	Non
149. **A-t-elle eu un test de dépistage du diabète (mesure de la glycémie après ingestion d'eau sucrée) ?**	☐ 1	☐ 2
150. **Avait-elle un "diabète de grossesse" (diabète gestationnel) ?**	☐ 1	☐ 2
151. **A-t-elle eu des insomnies du petit matin ?**	☐ 1	☐ 2
152. **A-t-elle pris des médicaments pour s'endormir ?**	☐ 1	☐ 2

153. **Au cours de sa grossesse, la mère a-t-elle pris des médicaments non encore mentionnés ?**

☐ 1 Oui ☐ 2 Non

154. **Une sage-femme est-elle venue au domicile pendant cette grossesse ?** ☐ 1 Oui ☐ 2 Non

© 2008-2016, INSERM U 1169

155. **Pour des raisons professionnelles, la mère a-t-elle rendu visite, à des personnes hospitalisées pendant cette grossesse ?**

☐ 1 Oui ☐ 2 Non

156. **Pour d'autres raisons, la mère a-t-elle rendu visite à une personne hospitalisée pendant cette grossesse ?**

☐ 1 Oui ☐ 2 Non

157. **Y a-t-il eu un autre événement de santé marquant pendant cette grossesse ?**

☐ 1 Oui ☐ 2 Non

L'accouchement

158. **Y a-t-il eu une menace d'accouchement prématuré ?**

158.1. ☐ 1 Oui **Si oui, la mère a-t-elle reçu un traitement pour cela ?** ☐ 1 Oui ☐ 2 Non

☐ 2 Non

**La mère a-t-elle :**	Oui	Non
159. **Pris des antibiotiques avant la naissance ?**	☐ 1	☐ 2
160. **Eu une rupture des membranes ?**	☐ 1	☐ 2
160.1. **Si oui, a-t-elle reçu un traitement pour cela ?** ☐ 1 Oui ☐ 2 Non		
161. **Eu une anesthésie générale pendant l'accouchement ?**	☐ 1	☐ 2
162. **Eu une anesthésie péridurale ?**	☐ 1	☐ 2

163. **Y a-t-il eu une révision utérine lors de l'accouchement ("main dans l'utérus") ?**

☐ 1 Oui ☐ 2 Non ☐ 3 Ne sait pas

164. **Comment le travail s'est-il déclenché ?**

☐ 1 Spontanément

164.1. ☐ 2 Avec des médicaments ?

Dans ce cas : ☐ 1 Le travail a été déclenché avec ocytocine par voie intraveineuse

☐ 2 Le travail a été déclenché par prostaglandines par voie vaginale

☐ 3 Autre ou ne sait pas

165. **La mère a-t-elle eu des antibiotiques pendant le travail ?**

☐ 1 Oui

☐ 2 Non

☐ 3 Ne sait pas

166. **La mère a-t-elle accouché ?**

166.1. ☐ 1 par voie basse

166.1.1. **Dans ce cas :** ☐ 1 Accouchement par forceps, ventouse

☐ 2 Accouchement spontané

☐ 2 par césarienne

167. **La mère a-t-elle eu des jumeaux ?**

167.1. ☐ 1 Oui **Si oui, les jumeaux étaient-il :** ☐ 1 Vrais ☐ 2 Faux ☐ 3 Ne sait pas

☐ 2 Non

© 2008-2016, INSERM U 1169

La mère après l'accouchement

168. **La mère a-t-elle eu des hémorragies génitales après l'accouchement ayant nécessité un traitement ?**

☐ 1 Oui ☐ 2 Non

169. **La mère a-t-elle pris des antibiotiques après la naissance ?** ☐ 1 Oui ☐ 2 Non

**La mère a-t-elle eu une supplémentation :**	Oui	Non	Ne sait pas
170. **En polyvitamines ?**	☐ 1	☐ 2	☐ 3
171. **En fer (Tardyferon, Fumafer, Ferrograd) ?**	☐ 1	☐ 2	☐ 3
172. **En calcium ?**	☐ 1	☐ 2	☐ 3
173. **En vitamine D (Uvédose) ?**	☐ 1	☐ 2	☐ 3
174. **En magnésium ?**	☐ 1	☐ 2	☐ 3

175. **Le bébé a-t-il été nourri par allaitement maternel, même mixte ou partiel ?**

☐ 1 Oui ☐ 2 Non ⇨ *Allez à la question 176*

**Si oui, la mère a-t-elle :**	Oui	Non
175.1. Pris une contraception orale pendant l'allaitement ?	☐ 1	☐ 2
175.2. Pris des antibiotiques pendant l'allaitement ?	☐ 1	☐ 2
175.3. Pris d'autres médicaments pendant l'allaitement ?	☐ 1	☐ 2
175.4. Mis de la crème sur les mamelons ?	☐ 1	☐ 2
175.5. Eu des fissures sur les mamelons ?	☐ 1	☐ 2

175.6. **Le bébé a-t-il été nourri exclusivement par allaitement maternel (au moins une période) ?**

175.6.1. ☐ 1 Oui **Si oui, quelle a été la durée de l'allaitement maternel exclusif ?**

☐ 1 moins d'un mois

☐ 2 entre un et trois mois

☐ 3 entre trois et six mois

☐ 4 plus de 6 mois

☐ 2 Non

2.4 - La famille autour de la mère pendant cette grossesse

Les autres membres de la famille

176. **Pendant cette grossesse, y avait-il un ou plusieurs autres petits enfants de moins de 6 ans à la maison ?**

☐ 1 Oui ☐ 2 Non ⇨ *Allez à la question 177 page 22*

176.1. **Si oui, cet ou ces enfants, allaient-ils en crèche ou à la maternelle pendant cette grossesse ?**

☐ 1 Oui ☐ 2 Non

176.2. **Un de ces enfants a-t-il été malade pendant cette grossesse ?**

176.2.1. ☐ 1 Oui **Si oui, a-t-il vu un médecin ?** ☐ 2 Oui ☐ 2 Non

176.2.1.1. **Si oui, y a-t-il eu un diagnostic ?** ☐ 1 Oui ☐ 2 Non

☐ 2 Non

☐ 3 Ne se souvient pas

© 2008-2016, INSERM U 1169

Sans toutefois vous livrer à une enquête trop poussée,
pouvez-vous porter une attention particulière aux questions suivantes ?

**Pendant cette grossesse, un enfant ou un adulte vivant à la maison a-t-il eu :**	Oui	Non
177. **La rougeole ?**	☐ 1	☐ 2
178. **Les oreillons ?**	☐ 1	☐ 2
179. **La varicelle ?**	☐ 1	☐ 2
180. **Un syndrome pied-main-bouche (vésicules sur les paumes, les plantes de pieds et dans la bouche) ?**	☐ 1	☐ 2
181. **La grippe ?**	☐ 1	☐ 2
182. **Une infection respiratoire ou une bronchiolite ?**	☐ 1	☐ 2
183. **Une hépatite ?**	☐ 1	☐ 2
184. **Des épisodes fébriles répétés ?**	☐ 1	☐ 2
185. **Une méningite virale ?**	☐ 1	☐ 2

186. **Un enfant ou un adulte vivant à la maison a-t-il eu une diarrhée aiguë avec des vomissements ?**

186.1. ☐ 1 Oui **Si oui, d'été ?** ☐ 1 Oui ☐ 2 Non
186.2. **d'hiver ?** ☐ 1 Oui ☐ 2 Non
☐ 2 Non
☐ 3 Ne se souvient pas

187. **Un médecin est-il venu au domicile pendant cette grossesse ?** ☐ 1 Oui ☐ 2 Non

188. **Une infirmière est-elle venue au domicile pendant cette grossesse ?** ☐ 1 Oui ☐ 2 Non

Sans toutefois vous livrer à une enquête trop poussée,
pouvez-vous porter une attention particulière aux questions suivantes ?

Pendant cette grossesse, vous rappelez-vous si <u>dans l'entourage</u> des parents ou des enfants, une personne a eu :

	**Oui**	**Non**	**Ne sait pas**
189. **La rougeole ?**	☐ 1	☐ 2	☐ 3
190. **Les oreillons ?**	☐ 1	☐ 2	☐ 3
191. **La varicelle ?**	☐ 1	☐ 2	☐ 3
192. **Un syndrome pied-main-bouche (vésicules sur les paumes, les plantes de pieds et dans la bouche) ?**	☐ 1	☐ 2	☐ 3
193. **Une infection respiratoire ou une bronchiolite ?**	☐ 1	☐ 2	☐ 3
194. **La grippe ?**	☐ 1	☐ 2	☐ 3
195. **Une hépatite ?**	☐ 1	☐ 2	☐ 3
196. **Des épisodes fébriles répétés ?**	☐ 1	☐ 2	☐ 3
197. **Une méningite virale ?**	☐ 1	☐ 2	☐ 3

198. **Vous rappelez-vous si <u>dans l'entourage</u> des parents ou des enfants, une personne a eu une diarrhée aiguë avec des vomissements ?**

198.1. ☐ 1 Oui **Si oui, d'été ?** ☐ 1 Oui ☐ 2 Non
198.2. **d'hiver ?** ☐ 1 Oui ☐ 2 Non
☐ 2 Non

© 2008-2016, INSERM U 1169

3 - Lorsque le futur diabétique était un bébé

*Aidez-vous du **carnet de santé** pour répondre aux questions suivantes !*

199. **Comment le bébé est-il né ?** ☐ 1 Normalement ☐ 2 Par le siège ☐ 2 Ne sait pas

200. **Le bébé est-il né à terme ?** ☐ 1 Oui ☐ 2 Non

**Le bébé a-t-il :**	Oui	Non
201. **Eu une “souffrance périnatale” (avec hospitalisation) ?**	☐ 1	☐ 2
202. **Reçu une perfusion peu après la naissance ?**	☐ 1	☐ 2
203. **Reçu un gavage alimentaire par sonde ?**	☐ 1	☐ 2
204. **Eu la jaunisse ?**	☐ 1	☐ 2
205. **Eu une infection néonatale ?**	☐ 1	☐ 2

206. **Le bébé a-t-il eu d’autres problèmes médicaux à la naissance ?** ☐ 1 Oui ☐ 2 Non

207. **Le bébé a-t-il eu des problèmes de peau ?**

207.1. ☐ 1 Oui **Si oui (plusieurs réponses possibles) :**
- ☐ 1 Eczéma ?
- ☐ 2 Boutons ou pustules surinfectées ?
- ☐ 3 Ni l’un ni l’autre

☐ 2 Non

Hygiène

	**Oui**	**Non**
208. **Le bébé était-il baigné et lavé au savon ou au gel ?**	☐ 1	☐ 2
209. **La mère lui mettait-elle des crèmes, des laits corporels ?**	☐ 1	☐ 2
210. **La mère utilisait-elle un mouche-bébé ?**	☐ 1	☐ 2
211. **Le bébé allait-il à la piscine (bébé nageur...) ?**	☐ 1	☐ 2

Alimentation

212. **Le bébé prenait-il un lait spécial sans protéines de lait de vache (allergie, intolérance) ?**

212.1. ☐ 1 Oui **Si oui, était-ce un lait aux protéines de soja ?** ☐ 1 Oui ☐ 2 Non

☐ 2 Non

213. **Avait-il changé plusieurs fois de marque de lait ?** ☐ 1 Oui ☐ 2 Non

214. **L’eau consommée par le bébé était l’eau du robinet exclusivement ?** ☐ 1 Oui ☐ 2 Non

215. **A quel âge a-t-il commencé à manger d’autres aliments que le lait (légumes ou fruits ou viande) ?**
- ☐ 1 avant 4 mois
- ☐ 2 entre 4 et 5 mois
- ☐ 3 entre 5 et 6 mois
- ☐ 4 après 6 mois

© 2008-2016, INSERM U 1169

216. **Le bébé consommait-il des <u>petits pots du commerce</u> ?**

216.1. ☐ 1 Oui **Si oui, en quelle quantité ?**

☐ 1 Plus de 4 par jour

☐ 2 Plus de 2 par jour

☐ 3 Moins de 2 petits pots par jour

☐ 2 Non

217. **Le bébé consommait-il des <u>petits pots de fruits du commerce</u> ?**

217.1. ☐ 1 Oui **Si oui, en quelle quantité ?**

☐ 1 Plus de 4 par jour

☐ 2 Plus de 2 par jour

☐ 3 Moins de 2 petits pots par jour

☐ 2 Non

218. **Le bébé consommait-il des <u>petits pots de fruits faits à la maison</u> ?**

218.1. ☐ 1 Oui **Si oui, en quelle quantité ?**

☐ 1 Plus de 4 par jour

☐ 2 Plus de 2 par jour

☐ 3 Moins de 2 petits pots par jour

☐ 2 Non

219. **Aimait-il particulièrement les petits pots contenant des aliments sucrés par rapports aux aliments non sucrés ?** ☐ 1 Oui ☐ 2 Non

220. **Le patient avait-il bon appétit, diriez-vous qu'il était :**

☐ 1 Gros mangeur

☐ 2 Normal

☐ 3 Petit mangeur

© 2008-2016, INSERM U 1169

4 - La vie médicale du futur diabétique

4.1 - Vie médicale du patient avant le diabète

Aidez-vous de votre mémoire et du carnet de santé : répondez “spontanément”, comme la mémoire vous revient, sans vous mettre sous pression pour fournir des réponses “parfaites”

221. **Le patient, bébé ou petit enfant avait-il eu une brûlure ?** ☐ 1 Oui ☐ 2 Non

222. **Le patient, bébé ou petit enfant avait-il eu un accident (chute) ?**

222.1. ☐ 1 Oui **Si oui : avec traumatisme crânien ?** ☐ 1 Oui ☐ 2 Non

222.2. **avec fracture osseuse ?** ☐ 1 Oui ☐ 2 Non

☐ 2 Non

Le patient a-t-il pris, entre sa naissance et la découverte de son diabète :

	Souvent<br>5 fois par an ou plus souvent	Assez souvent<br>3 à 4 fois par an	Rarement<br>1 ou 2 fois par an	Jamais
223. **Du paracétamol** (Doliprane, Efferalgan...), **de l’ibuprofen** (Advyl, Nurofen...) **ou de l’aspirine** (Aspegic...)	☐ 1	☐ 2	☐ 3	☐ 4
224. **Un anti-inflammatoire stéroïdien, un corticoïde :** Solupred, Celestene, Cortancyl	☐ 1	☐ 2	☐ 3	☐ 4
225. **Un antibiotique :** Amoxicilline, Clamoxil, Orelox, Augmentin, Zithromax...	☐ 1	☐ 2	☐ 3	☐ 4
226. **Un médicament contre les virus :** Zovirax, Tamiflu	☐ 1	☐ 2	☐ 3	☐ 4
227. **Un antifongique contre le muguet :** Daktarin, Mycostatine	☐ 1	☐ 2	☐ 3	☐ 4
228. **Un médicament contre les allergies :** Atarax, Polaramine, Primalan	☐ 1	☐ 2	☐ 3	☐ 4
229. **Un médicament contre la toux :** Toplexil, Tussilene, Mucomyst	☐ 1	☐ 2	☐ 3	☐ 4
230. **Un bronchodilatateur :** Ventoline, Serevent...	☐ 1	☐ 2	☐ 3	☐ 4
231. **Un médicament contre les brûlures d’estomac, l’acidité gastrique :** Mopral (Omeprazol), Ranitidine.	☐ 1	☐ 2	☐ 3	☐ 4
232. **Un médicament contre les maux de ventre :** Debridat...	☐ 1	☐ 2	☐ 3	☐ 4
233. **Un antidiarrhéique :** Ultralevure, Lactéol	☐ 1	☐ 2	☐ 3	☐ 4
234. **Un antidiarrhéique :** Imodium, Tiorfan...	☐ 1	☐ 2	☐ 3	☐ 4

235. **Le patient avait-il eu des poux ?** ☐ 1 Oui ☐ 2 Non

236. **Le patient avait-il eu des parasites digestifs (ascaris, oxyures) ?** ☐ 1 Oui ☐ 2 Non

© 2008-2016, INSERM U 1169

237. **Le patient était-il en contact avec la terre (jardin, bac à sable...) avant la survenue du diabète ?**

☐ 1 Tous les jours
☐ 2 Au moins une fois par semaine
☐ 3 Au moins une fois par mois
☐ 4 Plus rarement
☐ 5 Pendant les vacances seulement
☐ 6 Jamais

238. **Le patient avait-il été chez le dentiste avant la survenue du diabète ?**

238.1. ☐ 1 Oui **Si oui, combien de fois ?**

☐ 1 1 fois
☐ 1 2 fois
☐ 3 Plus de 2 fois

☐ 2 Non

239. **Combien de fois par jour se brossait-il les dents ?**

☐ 1 2 fois par jour
☐ 2 1 fois
☐ 3 Moins

240. **Le patient portait-il des lunettes ou/et des lentilles de correction de vue?**

240.1. ☐ 1 Oui **Si oui, quelle était son acuité visuelle <u>corrigée</u> ?**

☐ 1 10/10 ☐ 2 9/10 ☐ 3 8/10 ☐ 4 7/10 ☐ 5 moins de 6/10

☐ 2 Non

241. **Le patient était-il myope ?**

☐ 1 Oui **Si oui, quelle est la puissance de sa correction ?**

		De - 1 à - 3 dioptries	De - 3 à - 6 dioptries	Plus forte que - 6 dioptries
241.1.	Œil droit	☐ 1	☐ 2	☐ 3
241.2.	Œil gauche	☐ 1	☐ 2	☐ 3

☐ 2 Non

242. **Diriez-vous que le futur diabétique était :**

☐ 1 “Tout le temps malade” (rhumes, rhinopharyngites, etc)
☐ 2 Ou plutôt “jamais malade”
☐ 3 Ni l’un ni l’autre

4.2 - Maladies infectieuses du patient avant le diagnostic du diabète

Les questions qui suivent concernent les vaccins, aidez-vous du ***carnet de santé*** *!*

243. **Le vaccin combiné DTCPH (Infanrix Quinta, Pentacoc, Pentavac, Tetravac acellulaire...) a été fait :**

☐ 1 A la date normale
☐ 2 Retardé par de la fièvre
☐ 3 Non fait
☐ 4 Ne sait pas

244. **Le vaccin contre les pneumocoques Prévenar a-t-il été fait à deux mois (s’il a été fait après l’âge de deux mois répondez «Non») ?** ☐ 1 Oui ☐ 2 Non ☐ 3 Ne sait pas

© 2008-2016, INSERM U 1169

245. **Le vaccin hépatite B (Engerix, Genhevac B, Hbvaxpro 5...) a-t-il été fait à deux mois (s'il a été fait après l'âge de deux mois répondez «Non») ?** ☐ 1 Oui ☐ 2 Non ☐ 3 Ne sait pas

**Le patient a-t-il eu avant le diagnostic du diabète :**	Oui	Non	Ne sait pas
246. **Le vaccin ROR (rougeole-oreillons-rubéole) ?**	☐ 1	☐ 2	☐ 3
247. **Le vaccin de l'hépatite A (Havrix, Twinrix, Avaxim, Tyavax...) ?**	☐ 1	☐ 2	☐ 3
248. **Le vaccin contre la fièvre jaune (Stamaril...) ?**	☐ 1	☐ 2	☐ 3
249. **Le vaccin « méningocoques » (Menínvact, Memomune, Vaccin méningococcique A + C...) ?**	☐ 1	☐ 2	☐ 3
250. **Un autre vaccin de voyageur (typhoïde, encéphalite à tiques...) ?**	☐ 1	☐ 2	☐ 3

Sans toutefois vous livrer à une enquête trop poussée, pouvez-vous porter une attention particulière aux questions suivantes qui concernent des virus qui ont été incriminés dans le diabète ?

**Le patient, avant le diagnostic du diabète a-t-il eu :**	Oui	Non	Ne sait pas
251. **La roséole ou exanthème subit, la 5e maladie ?**	☐ 1	☐ 2	☐ 3
252. **Le patient a-t-il eu une éruption fébrile (boutons ou plaques rouges avec de la fièvre) non expliquée ?**	☐ 1	☐ 2	☐ 3
253. **Etait-il arrivé au patient de se mettre à vomir plusieurs fois, par exemple pendant une demi-journée, sans raison apparente ?**	☐ 1	☐ 2	☐ 3
254. **Un syndrome pied-main-bouche (éruption des paumes et des plantes et bulles dans la bouche) ?**	☐ 1	☐ 2	☐ 3
255. **Une infection respiratoire ou une bronchiolite ?**	☐ 1	☐ 2	☐ 3
256. **Le patient a-t-il eu une pancréatite hospitalisée ?**	☐ 1	☐ 2	☐ 3
257. **Une hépatite ?**	☐ 1	☐ 2	☐ 3
258. **Des épisodes fébriles répétés ?**	☐ 1	☐ 2	☐ 3
259. **Une méningite virale ?**	☐ 1	☐ 2	☐ 3

260. **Le patient a-t-il eu une diarrhée aiguë avec des vomissements ?**

☐ 1 Oui **Si oui :**

	Oui	Non	Ne sait pas
260.1. **durant plus de 5 jours avec fièvre ?**	☐ 1	☐ 2	☐ 3
260.2. **d'hiver ?**	☐ 1	☐ 2	☐ 3
260.3. **d'été ?**	☐ 1	☐ 2	☐ 3
260.4. **dans un contexte de diarrhée familiale ?**	☐ 1	☐ 2	☐ 3

☐ 2 Non

••••••••••••••••••

261. **Avant le diagnostic du diabète, le patient est-il allé à la crèche ?**

261.1. ☐ 1 Oui **Si oui, y a-t-il eu une méningite chez un autre enfant de la crèche ?**

☐ 1 Oui ☐ 2 Non ☐ 3 Ne sait pas

261.2. **y a-t-il eu une autre épidémie (maladie contagieuse) dans la crèche ?**

☐ 1 Oui ☐ 2 Non ☐ 3 Ne sait pas

☐ 2 Non

© 2008-2016, INSERM U 1169

262. **Avant le diagnostic du diabète, le patient est-il allé à la maternelle ?**

262.1. ☐ 1 Oui **Si oui, y a-t-il eu une méningite chez un autre enfant de la maternelle ?**

☐ 1 Oui ☐ 2 Non ☐ 3 Ne sait pas

262.2. **y a-t-il eu une autre épidémie (maladie contagieuse) dans la maternelle ?**

☐ 1 Oui ☐ 2 Non ☐ 3 Ne sait pas

☐ 2 Non

263. **Le patient avait-il été mordu par un animal ?**

263.1. ☐ 1 Oui **Si oui : lequel ?** ☐ 1 Un chat ☐ 2 Un chien ☐ 3 Un autre animal

☐ 2 Non

264. **Le patient avait-il été vacciné contre la rage ?** ☐ 1 Oui ☐ 2 Non

265. **Le patient avait-il déjà été griffé par un animal ?**

265.1. ☐ 1 Oui **Si oui : lequel ?** ☐ 1 Un chat ☐ 2 Un autre animal

☐ 2 Non

266. **Le patient avait-il tendance à être piqué par les moustiques ?** ☐ 1 Oui ☐ 2 Non

267. **Avait-il été mordu par une araignée ayant laissé une marque nette (marque rouge douloureuse ou non) ?** ☐ 1 Oui ☐ 2 Non

268. **Avait-il été mordu par une tique ayant laissé une marque nette (marque rouge douloureuse ou non) ?**

☐ 1 Oui ☐ 2 Non

269. **Avait-il été piqué par un animal ayant laissé une marque nette (marque rouge douloureuse ou non) ?**

269.1. ☐ 1 Oui **Si oui, lequel ?**

☐ 1 Une guêpe ☐ 2 Une abeille ☐ 3 Un autre insecte ☐ 4 Un poisson

☐ 2 Non

270. **Le patient avait-t-il été protégé par des traitements répulsifs anti-moustiques (crèmes, spray...) ?**

☐ 1 Oui ☐ 2 Non

271. **Le patient avait-t-il été traité avec des pommades aux corticoïdes ?** ☐ 1 Oui ☐ 2 Non

**Le patient a-t-il eu une conjonctivite (oeil rouge, oeil sale) :**	Oui	Non	Ne sait pas
272. **Immédiatement après la naissance ?**	☐ 1	☐ 2	☐ 3
273. **A distance de la naissance ?**	☐ 1	☐ 2	☐ 3
274. **Le patient a-t-il été traité par un collyre ?**	☐ 1	☐ 2	☐ 3

275. **Dans sa première année, le patient a-t-il eu au moins une infection ORL : otite, rhinite, rhinopharyngite...?**

☐ 1 Non ☐ 2 1 ou 2 ☐ 3 Plus de 2 ☐ 4 Ne sait pas

276. **Dans sa première année, le patient a-t-il eu des crises de toux ?**

276.1. ☐ 1 Oui **Si oui, a-t-il toussé avec des rejets ?** ☐ 1 Oui ☐ 2 Non

☐ 2 Non

☐ 3 Ne sait pas

© 2008-2016, INSERM U 1169

Entre sa naissance et le diagnostic du diabète, le patient :	Oui	Non	Ne sait pas
277. **A-t-il eu souvent le nez qui coulait ?**	☐ 1	☐ 2	☐ 3
278. **A-t-il eu un gros rhume avec fièvre pendant plus de 2 jours ?**	☐ 1	☐ 2	☐ 3
279. **Ronflait-il la nuit ?**	☐ 1	☐ 2	☐ 3
280. **A-t-il été opéré des végétations ?**	☐ 1	☐ 2	☐ 3
281. **A-t-il été opéré des amygdales ?**	☐ 1	☐ 2	☐ 3

282. **Entre sa naissance et le diagnostic du diabète, le patient a-t-il eu des angines ?**

282.1. ☐ 1 Oui **Si oui, le patient a-t-il eu des angines avec test de dépistage du streptocoque ?**

☐ 1 Oui ☐ 2 Non

282.2. **le patient a-t-il eu des angines traitées avec des antibiotiques ?**

☐ 1 Oui ☐ 2 Non

☐ 2 Non

☐ 3 Ne sait pas

Entre sa naissance et le diagnostic du diabète :	Oui	Non	Ne sait pas
283. **Le patient a-t-il eu des paracentèses (otites) ?**	☐ 1	☐ 2	☐ 3
284. **Lui a-t-on posé des yoyos dans les oreilles ?**	☐ 1	☐ 2	☐ 3
285. **Le patient a-t-il eu une laryngite ?**	☐ 1	☐ 2	☐ 3
286. **Le patient a-t-il eu des ganglions autour de la gorge (au niveau du cou) ?**	☐ 1	☐ 2	☐ 3
287. **Le patient a-t-il eu l'asthme du nourrisson ?**	☐ 1	☐ 2	☐ 3
288. **Le patient a-t-il eu une radio des poumons ?**	☐ 1	☐ 2	☐ 3

Le patient a-t-il eu :	Oui	Non	Ne sait pas
289. **Un traitement des diarrhées par Smecta ?**	☐ 1	☐ 2	☐ 3
290. **Des antidiarrhéiques ?**	☐ 1	☐ 2	☐ 3
291. **Des antibiotiques pour la diarrhée ?**	☐ 1	☐ 2	☐ 3
292. **Une analyse de germes de virus dans les selles, une coproculture ?**	☐ 1	☐ 2	☐ 3
293. **Des douleurs abdominales («coliques», cris) ?**	☐ 1	☐ 2	☐ 3
294. **Le patient a-t-il eu des infections urinaires ?**	☐ 1	☐ 2	☐ 3

295. **Le patient a-t-il été circoncis ?** ☐ 1 Oui ☐ 2 Non ☐ 3 Non applicable

296. **Le patient a-t-il eu d'autres maladies infectieuses ?**

296.1. ☐ 1 Oui **Si oui, plutôt à quelle saison ?**

☐ 1 Printemps

☐ 2 Eté

☐ 3 Automne

☐ 4 Hiver

☐ 5 N'importe quand

☐ 2 Non

© 2008-2016, INSERM U 1169

5 - Les habitudes de vie et l'environnement du futur diabétique

5.1 - Alimentation du patient avant la découverte du diabète

297. **Le patient aimait-il le sucre ?** ☐ 1 Beaucoup ☐ 2 Un peu ☐ 3 Pas du tout

298. **Le patient aimait-il le sel ?** ☐ 1 Beaucoup ☐ 2 Un peu ☐ 3 Pas du tout

Viandes

299. **A quel âge le patient a-t-il commencé à manger de la viande de boucherie ?**
☐ 1 Avant 6 mois ☐ 2 Entre 6 mois et un an ☐ 3 Après un an ☐ 4 Ne sait pas

**Avant le diagnostic du diabète le patient consommait-il :**	Oui	Non	Ne sait pas
300. **Du bœuf ?**	☐ 1	☐ 2	☐ 3
301. **Du mouton ?**	☐ 1	☐ 2	☐ 3
302. **De la volaille ?**	☐ 1	☐ 2	☐ 3
303. **Du poulet ?**	☐ 1	☐ 2	☐ 3
304. **Du lapin ?**	☐ 1	☐ 2	☐ 3
305. **Du porc ?**	☐ 1	☐ 2	☐ 3
306. **Du cheval ?**	☐ 1	☐ 2	☐ 3
307. **De la charcuterie froide (pâté, saucisson, jambon) achetée <u>en grande ou petite surface</u> ?**	☐ 1	☐ 2	☐ 3
308. **De la charcuterie type saucisse ou boudin achetée en <u>boucherie-charcuterie</u> ?**	☐ 1	☐ 2	☐ 3
309. **De la charcuterie type <u>saucisse industrielle</u> ?**	☐ 1	☐ 2	☐ 3
310. **De la charcuterie <u>préparée à la maison ou familiale</u> ?**	☐ 1	☐ 2	☐ 3
311. **Du gibier (lièvre, sanglier, cerf) ?**	☐ 1	☐ 2	☐ 3
312. **Du gibier à plumes (faisan, perdreaux, caille, etc) ?**	☐ 1	☐ 2	☐ 3

Poissons, coquillages, crustacés

313. **Avec quelle fréquence le patient consommait-il du poisson frais ?**
☐ 1 Plusieurs fois par semaine
☐ 2 Au moins une fois par semaine
☐ 3 Au moins une fois par mois
☐ 4 Plus rarement
☐ 5 Jamais ⇨ *Allez directement à la question 316 page 31*

314. **Le patient consommait-il du <u>poisson de rivière frais</u> ?**
☐ 1 Plusieurs fois par semaine
☐ 2 Au moins une fois par semaine
☐ 3 Au moins une fois par mois
☐ 4 Plus rarement
☐ 5 Jamais ⇨ *Allez directement à la question 315 page 31*

314.1. **Le consommait-il parfois cru ?** ☐ 1 Oui ☐ 2 Non

314.2. **Le patient consommait-il de la truite d'élevage ?** ☐ 1 Oui ☐ 2 Non

© 2008-2016, INSERM U 1169

315. **Le patient consommait-il du <u>poisson de mer frais</u> (colin, merlan, sole, lotte, limande, mulet, bar, etc) ?**

☐ 1 Plusieurs fois par semaine
☐ 2 Au moins une fois par semaine
☐ 3 Au moins une fois par mois
☐ 4 Plus rarement
☐ 5 Jamais ⇨ *Allez directement à la question 316*

315.1. **Le consommait-il parfois cru ?** ☐ 1 Oui ☐ 2 Non
315.2. **Le patient consommait-il du saumon frais ?** ☐ 1 Oui ☐ 2 Non

316. **Le patient consommait-il du poisson fumé (saumon, truite, anguille...) ?** ☐ 1 Oui ☐ 2 Non

317. **Le patient consommait-il du poisson en boîte (sardines, thon, maquereau...) ?**

☐ 1 Plusieurs fois par semaine
☐ 2 Au moins une fois par semaine
☐ 3 Au moins une fois par mois
☐ 4 Plus rarement
☐ 5 Jamais

318. **Le patient consommait-il des préparations de poisson type nuggetts, poisson pané, surimi... ?**

☐ 1 Au moins une fois par semaine
☐ 2 Au moins une fois par mois
☐ 3 Plus rarement
☐ 4 Jamais

319. **Le patient consommait-il des coquillages (huîtres, moules, bigorneaux...) ?**

☐ 1 Au moins une fois par semaine
☐ 2 Au moins une fois par mois
☐ 3 Plus rarement
☐ 4 Jamais

320. **Le patient consommait-il des crustacés (crabe, crevettes, homard...) ?**

☐ 1 Au moins une fois par semaine
☐ 2 Au moins une fois par mois
☐ 3 Plus rarement
☐ 4 Jamais

Légumes

321. **Le patient consommait-il des légumes venant d'une grande ou petite surface ?** ☐ 1 Oui ☐ 2 Non

322. **Le patient consommait-il des légumes venant d'une ferme d'un maraîcher ou de plantation familiale ?**

☐ 1 Oui ☐ 2 Non

323. **Le patient consommait-il des purées "<u>toutes prêtes</u>" à reconstituer (purée en flocons...) ?**

☐ 1 Plusieurs fois par semaine
☐ 2 Au moins une fois par semaine
☐ 3 Au moins une fois par mois
☐ 4 Plus rarement
☐ 5 Jamais

© 2008-2016, INSERM U 1169

324. **Les légumes étaient-ils épluchés et préparés à la maison ?**

☐ 1 Plusieurs fois par semaine
☐ 2 Au moins une fois par semaine
☐ 3 Au moins une fois par mois
☐ 4 Plus rarement
☐ 5 Jamais

325. **Le patient consommait-il des champignons cultivés (champignons de Paris, pleurotes...) ?**

☐ 1 Oui ☐ 2 Non

326. **Le patient consommait-il des champignons sauvages ?** ☐ 1 Oui ☐ 2 Non

Fruits

**Le patient consommait :**	Oui	Non
327. **Des fruits venant d'une grande ou petite surface ?**	☐ 1	☐ 2
328. **Des fruits venant d'une ferme ou d'un verger familial ?**	☐ 1	☐ 2
329. **Des bananes ?**	☐ 1	☐ 2
330. **Des litchis ou ananas, mangues, goyaves, papayes, fruits de la passion frais?**	☐ 1	☐ 2
331. **D'autres fruits exotiques frais ?**	☐ 1	☐ 2

Produits laitiers

332. **Le patient mangeait-il du fromage ?**

332.1. ☐ 1 Oui **Si oui, du fromage de chèvre ?** ☐ 1 Oui ☐ 2 Non
332.2. **du fromage de brebis ?** ☐ 1 Oui ☐ 2 Non
332.3. **du fromage de vache ?** ☐ 1 Oui ☐ 2 Non
☐ 2 Non

**Le patient consommait-il :**	Oui	Non
333. **Des laitages ou du fromage au lait cru ?**	☐ 1	☐ 2
334. **Habituellement les croûtes de fromage ?**	☐ 1	☐ 2
335. **Des oeufs frais ?**	☐ 1	☐ 2

336. **Le patient consommait des yaourts ou des petits-suisses ?**

☐ 1 Plusieurs fois par jour
☐ 2 Au moins une fois par jour
☐ 3 Au mois une fois par semaine
☐ 3 Au moins une fois par mois
☐ 4 Plus rarement
☐ 5 Jamais

© 2008-2016, INSERM U 1169

Dessert

Le patient consommait-il :	Plusieurs fois par jour	Au moins une fois par jour	Au moins une fois par semaine	Plus rarement	Jamais
337. Des gâteaux, biscuits	☐ 1	☐ 2	☐ 3	☐ 4	☐ 5
338. Des confitures	☐ 1	☐ 2	☐ 3	☐ 4	☐ 5
339. De la Marmite, de la Vegemite ou du Cenovis	☐ 1	☐ 2	☐ 3	☐ 4	☐ 5
340. Des confiseries du commerce (bonbons, fraises artificielles, etc) ?	☐ 1	☐ 2	☐ 3	☐ 4	☐ 5
341. Du chocolat ?	☐ 1	☐ 2	☐ 3	☐ 4	☐ 5
342. Des produits type Nutella ?	☐ 1	☐ 2	☐ 3	☐ 4	☐ 5

343. **Le patient consommait-il des cacahuètes ou des chips d'apéritif à la cacahuète de temps en temps ?**

☐ 1 Oui ☐ 2 Non

Huiles

Quels types d'huiles utilisait-on :	Oui	Non
344. De l'huile de tournesol ?	☐ 1	☐ 2
345. De l'huile d'olive ?	☐ 1	☐ 2
346. De l'huile de colza ?	☐ 1	☐ 2
347. De l'huile d'arachide ?	☐ 1	☐ 2
348. Des huiles mélangées (Isio4...) ?	☐ 1	☐ 2
349. D'autres types d'huiles ?	☐ 1	☐ 2

Boissons

Le patient consommait-il :	Plusieurs fois par jour	Au moins une fois par jour	Au moins une fois par semaine	Plus rarement	Jamais
350. Des jus de fruits ?	☐ 1	☐ 2	☐ 3	☐ 4	☐ 5
351. Du Coca-Cola ou du Pepsi ?	☐ 1	☐ 2	☐ 3	☐ 4	☐ 5
352. Des sodas gazeux ?	☐ 1	☐ 2	☐ 3	☐ 4	☐ 5
353. D'autres boissons sucrées ?	☐ 1	☐ 2	☐ 3	☐ 4	☐ 5

Modes de cuisson et de conservation

354. **La cuisine familiale utilisait un four à micro-ondes pour cuire ou réchauffer les aliments ?**

☐ 1 Tous les jours
☐ 2 Au moins une fois par semaine
☐ 3 Au moins une fois par mois
☐ 4 Plus rarement
☐ 5 Jamais

© 2008-2016, INSERM U 1169

**Le patient consommait-il :**	Oui	Non
355. **Des viandes surgelées ?**	☐ 1	☐ 2
356. **Des poissons surgelés ?**	☐ 1	☐ 2
357. **Des légumes surgelés ?**	☐ 1	☐ 2

358. **Y avait-il des congélations familiales ?** ☐ 1 Oui ☐ 2 Non ⇨ *Allez directement à la question 359*

**Si oui, y avait-il des congélations familiales :**	Oui	Non
358.1. **De viande ?**	☐ 1	☐ 2
358.2. **De poisson ?**	☐ 1	☐ 2
358.3. **De légumes ?**	☐ 1	☐ 2
358.4. **De gibier ?**	☐ 1	☐ 2

**Le patient consommait-il :**	Oui	Non
359. **Des conserves familiales de légumes ?**	☐ 1	☐ 2
360. **Des conserves de fruits ?**	☐ 1	☐ 2
361. **Des conserves de produits animaux (pâté, couscous, cassoulet, thon...)?**	☐ 1	☐ 2

5.2 - Habitat du patient avant la découverte du diabète

Toutes les questions qui suivent concernent l'habitation dans laquelle le patient passait le plus de temps

362. **Quelle était approximativement la surface de l'habitation ?**

☐ 1 moins de 40 m²
☐ 2 de 40 m² à moins de 70 m²
☐ 3 de 70 m² à moins de 100 m²
☐ 4 de 100 m² à moins de 150 m²
☐ 5 150 m² ou plus

363. **Comment était chauffée cette habitation ?**

☐ 1 Chauffage individuel (différent pour chaque pièce)
☐ 2 Chauffage central

364. **Quel était le mode de chauffage (plusieurs réponses possibles) ?**

☐ 1 Chauffage électrique (convecteurs, radiateurs électriques...)
☐ 2 Chauffage au gaz
☐ 3 Chauffage au fioul
☐ 4 Cheminée
☐ 5 Autre

© 2008-2016, INSERM U 1169

**Dans l'habitation, utilisait-on :**	Oui	Non
365. **Des produits contre les souris, rats ?**	☐ 1	☐ 2
366. **Des produits contre les fourmis ?**	☐ 1	☐ 2
367. **Des produits contre les cafards ?**	☐ 1	☐ 2
368. **Des produits (plaquettes...) anti-mouches ?**	☐ 1	☐ 2
369. **Des produits anti-moustiques (prise...) ?**	☐ 1	☐ 2
370. **Des produits antimites ?**	☐ 1	☐ 2

371. **De quel type d'habitation s'agissait-il ?**

☐ 1 Une maison

371.1. **Dans ce cas à quel étage se situait la chambre du patient ?**

☐ 1 Au rez-de-chaussée
☐ 2 Au 1er, 2e, 3e ou 4e étage
☐ 3 Plus haut

371.2. **L'évacuation des eaux usées se faisait-elle ?**

☐ 1 Dans une fosse septique
☐ 2 Dans le tout-à-l'égout

☐ 2 Un appartement

371.3. **Dans ce cas à quel étage se situait l'appartement ?**

☐ 1 Au rez-de-chaussée
☐ 2 Au 1er, 2e, 3e ou 4e étage
☐ 3 Au 5e, 6e, 7e ou 8e étage
☐ 4 Au 9e étage ou plus haut

☐ 3 Une caravane

372. **Y avait-il un jardin ou un terrain autour de l'habitation ?**

☐ 1 Oui
☐ 2 Non ⇨ *Allez directement à la question 373 page 36*

**Dans le jardin :**	Oui	Non
372.1. **Y avait-il des fleurs ?**	☐ 1	☐ 2
372.2. **Y avait-il des légumes ?**	☐ 1	☐ 2
372.3. **Y avait-il des arbres fruitiers, des fruits ?**	☐ 1	☐ 2

372.4. **Le patient jouait-il dans ce jardin ou ce terrain ?**

☐ 1 Tous les jours
☐ 2 Au moins une fois par semaine
☐ 3 Au moins une fois par mois
☐ 4 Plus rarement
☐ 5 Jamais

© 2008-2016, INSERM U 1169

**Utilisait-on dans le jardin :**	Oui	Non
372.5. **Des engrais (naturels ou chimiques) ?**	☐ 1	☐ 2
372.6. **Des insecticides (pour lutter contre les insectes) ?**	☐ 1	☐ 2
372.7. **Des fongicides (pour lutter contre les champignons) ?**	☐ 1	☐ 2
372.8. **Des désherbants (pour lutter contre les mauvaises herbes) ?**	☐ 1	☐ 2
372.9. **Des produits pour lutter contre les escargots et limaces ?**	☐ 1	☐ 2
372.10. **Des produits contre les fourmis ?**	☐ 1	☐ 2
372.11. **Des produits pour lutter contre des animaux nuisibles : taupes, souris... ?**	☐ 1	☐ 2

372.12. **Y avait-il une piscine dans le jardin ?** ☐ 1 Oui ☐ 2 Non

La chambre du patient

373. **Le patient avait-il une chambre pour lui seul ?**

☐ 1 Oui

373.1. ☐ 2 Non **Si non, partageait-il une chambre avec ses frères ou sœurs ?** ☐ 1 Oui ☐ 2 Non

373.2. **partageait-il une chambre avec ses parents ?** ☐ 1 Oui ☐ 2 Non

374. **Le sol de la chambre du patient était en :**

☐ 1 Moquette
☐ 2 Carrelage
☐ 3 Parquet
☐ 4 Autre

375. **La literie (oreillers ou couettes) du patient était-elle en plumes naturelles ?** ☐ 1 Oui ☐ 2 Non

**Utilisait-on dans la chambre du patient :**	Oui	Non
376. **Des produits contre les fourmis ?**	☐ 1	☐ 2
377. **Des produits (plaquettes...) anti-mouches ?**	☐ 1	☐ 2
378. **Des produits anti-moustiques (prise...) ?**	☐ 1	☐ 2
379. **Des produits antimites ?**	☐ 1	☐ 2
380. **Des produits contre les souris, rats ?**	☐ 1	☐ 2

381. **La chambre du patient avait une/des fenêtre(s) sur** (plusieurs réponses possibles) **:**

☐ 1 Rue
☐ 2 Jardin
☐ 3 Cour intérieure

382. **L'exposition principale de la fenêtre de la chambre était :**

☐ 1 Ouest
☐ 2 Est
☐ 3 Sud
☐ 4 Nord

383. **De la fenêtre de la chambre voyait-on des arbres ?** ☐ 1 Oui ☐ 2 Non

384. **De la fenêtre de la chambre voyait-on des champs ?** ☐ 1 Oui ☐ 2 Non

© 2008-2016, INSERM U 1169

Le reste de l'habitation

Dans la maison :	Oui	Non
385. La salle de bain du patient était-elle séparée de celle des parents ?	☐ 1	☐ 2
386. Les toilettes WC du patient étaient-elles séparées de celles des parents ?	☐ 1	☐ 2
387. Les toilettes WC étaient-elles dans la salle de bain du patient ?	☐ 1	☐ 2
388. Utilisait-on une machine à laver la vaisselle pour la vaisselle familiale ?	☐ 1	☐ 2
389. Utilisait-on un aspirateur ?	☐ 1	☐ 2
390. Y avait-il eu des travaux de peinture dans la maison ou l'appartement ?	☐ 1	☐ 2
391. Y avait-il eu d'autres travaux ?	☐ 1	☐ 2

392. **Avec quelle fréquence le réfrigérateur était-il nettoyé à fond ?**

☐ 1 Au moins une fois par semaine
☐ 2 Au moins une fois par mois
☐ 3 Au moins une fois par trimestre
☐ 4 Au moins une fois par an
☐ 5 Moins souvent

393. **Y avait-il des plantes en pots à la maison ou sur un balcon ?** ☐ 1 Oui ☐ 2 Non

394. **Y avait-il des fleurs dans des vases avec de l'eau ?** ☐ 1 Oui ☐ 2 Non

Autour de l'habitation

395. **De l'habitation, voyait-on des arbres ?** ☐ 1 Oui ☐ 2 Non

396. **De l'habitation, voyait-on des champs ?** ☐ 1 Oui ☐ 2 Non

397. **A proximité de l'habitation, y avait-il des terres agricoles ?**

☐ 1 Oui ☐ 2 Non ⇨ *Allez à la question 398*

Si oui, y avait-il des cultures :	Oui	Non	Ne sait pas
397.1. De blé ?	☐ 1	☐ 2	☐ 3
397.2. De maïs ?	☐ 1	☐ 2	☐ 3
397.3. De vigne ?	☐ 1	☐ 2	☐ 3
397.4. De colza ?	☐ 1	☐ 2	☐ 3
397.5. De tournesol ?	☐ 1	☐ 2	☐ 3
397.6. De légumes ?	☐ 1	☐ 2	☐ 3

A proximité de l'habitation, y avait-il :	Oui	Non
398. Un élevage intensif de volailles ?	☐ 1	☐ 2
399. Un élevage intensif de porcs ?	☐ 1	☐ 2
400. Une usine ?	☐ 1	☐ 2

© 2008-2016, INSERM U 1169

5.3 - Le patient est gardé avant la découverte du diabète

401. **Le patient avant trois ans était-il gardé ?** ☐ 1 Oui ☐ 2 Non ⇨ *Allez à la question 402 page 40*

Sans toutefois vous livrer à une enquête trop poussée, pouvez-vous porter une attention particulière aux questions suivantes ?

	**Un enfant ou une personne gardant le patient, futur diabètique a-t-il eu :**	Oui	Non
401.1.	**La rougeole ?**	☐ 1	☐ 2
401.2.	**Les oreillons ?**	☐ 1	☐ 2
401.3.	**La varicelle ?**	☐ 1	☐ 2
401.4.	**Un syndrome pied main bouche (éruption des paumes et des plantes et bulles dans la bouche) ?**	☐ 1	☐ 2
401.5.	**La grippe ?**	☐ 1	☐ 2
401.6.	**Une infection respiratoire ou une bronchiolite ?**	☐ 1	☐ 2
401.7.	**Une diarrhée aiguë avec des vomissements ?**	☐ 1	☐ 2
401.8.	**Une hépatite ?**	☐ 1	☐ 2
401.9.	**Des épisodes fébriles répétés ?**	☐ 1	☐ 2
401.10.	**Une méningite virale ?**	☐ 1	☐ 2

••••••••••••••••••

401.11. **Avant trois ans (et avant le diagnostic du diabète), le patient était-il gardé en crèche ?**
☐ 1 Oui ☐ 2 Non ⇨ *Allez à la question 401.12*

	**Si oui, allait-il à la crèche**	**Oui**	**Non**
401.11.1.	**Entre 3 et 6 mois ?**	☐ 1	☐ 2
401.11.2.	**Entre 6 mois et un an ?**	☐ 1	☐ 2
401.11.3.	**Entre 1 an et 2 ans ?**	☐ 1	☐ 2
401.11.4.	**Entre 2 ans et 3 ans ?**	☐ 1	☐ 2
401.11.5.	**Allait-il en crèche collective ?**	☐ 1	☐ 2
401.11.6.	**Allait-il en crèche familiale ?**	☐ 1	☐ 2
401.11.7.	**Allait-il en garde partagée ?**	☐ 1	☐ 2

401.12. **Avant trois ans, le patient était-il gardé chez une nourrice ?**
☐ 1 Oui ☐ 2 Non ⇨ *Allez à la question 402 page 40*

401.12.1. **Y avait-il d'autres enfants chez cette nourrice ?** ☐ 1 Oui ☐ 2 Non

401.12.2. **Quelle était approximativement la surface de l'habitation de la nourrice ?**
- ☐ 1 moins de 40 m²
- ☐ 2 de 40 m² à moins de 70 m²
- ☐ 3 de 70 m² à moins de 100 m²
- ☐ 4 de 100 m² à moins de 150 m²
- ☐ 5 150 m² ou plus

401.12.3. **Le patient avait-il une chambre pour lui tout seul ?** ☐ 1 Oui ☐ 2 Non

© 2008-2016, INSERM U 1169

401.12.4. **Le sol de la chambre du patient était en :**

☐ 1 Moquette

☐ 2 Carrelage

☐ 3 Parquet

☐ 4 Autre

401.12.5. **La chambre du patient avait une fenêtre sur :**

☐ 1 Rue

☐ 2 Jardin

☐ 3 Cour intérieure

401.12.6. **Voyait-on des arbres de l'habitation ou de la fenêtre de la chambre ?**

☐ 1 Oui ☐ 2 Non

401.12.7. **Voyait-on des champs de l'habitation ou de la fenêtre de la chambre ?**

☐ 1 Oui ☐ 2 Non

401.12.8. **Dans quel type d'habitation la nourrice recevait-elle les enfants ?**

401.12.8.1. ☐ 1 Une maison : **Dans ce cas, à quel étage était la chambre du patient ?**

☐ 1 Au rez-de-chaussée

☐ 2 Au 1er, 2e, 3e ou 4e étage

☐ 3 Plus haut

401.12.8.2. ☐ 1 Un appartement : **Dans ce cas, à quel étage se situait l'appartement ?**

☐ 1 Au rez-de-chaussée

☐ 2 Au 1er, 2e, 3e ou 4e étage

☐ 3 Au 5e, 6e, 7e ou 8e étage

☐ 4 Au 9e étage ou plus haut

401.12.9. **Y avait-il un jardin ou un terrain autour de l'habitation de la nourrice ?**

☐ 1 Oui ☐ 2 Non ⇨ *Allez à la question 402 page 40*

	**Dans le jardin ou le terrain :**	Oui	Non
401.12.9.1.	**Y avait-il des fleurs ?**..	☐ 1	☐ 2
401.12.9.2.	**Y avait-il des légumes ?**....................................	☐ 1	☐ 2
401.12.9.3.	**Y avait-il des fruits ?**..	☐ 1	☐ 2

401.12.9.4. **Le patient jouait-il dans ce jardin ou terrain ?**

☐ 1 Tous les jours

☐ 2 Au moins une fois par semaine

☐ 3 Au moins une fois par mois

☐ 4 Plus rarement

☐ 4 Jamais

401.12.9.5. **Y avait-il une piscine dans le jardin ?** ☐ 1 Oui ☐ 2 Non

© 2008-2016, INSERM U 1169

5.4 - Les déplacements, loisirs du patient avant la découverte du diabète

402. **Le futur diabétique allait-il au jardin public, au square étant petit ?**

402.1. ☐ 1 Oui **Si oui, jouait-il dans un bac à sable dans ce jardin public ou ce square ?**

☐ 1 Oui ☐ 2 Non

☐ 2 Non

Ecole

Le patient a-t-il été :

403. **A l'école maternelle avant le diabète ?** ☐ 1 Oui ☐ 2 Non

404. **A l'école primaire avant le diabète ?** ☐ 1 Oui ☐ 2 Non

Si le patient allait à l'école ou à la crèche avant le diabète, comment se déplaçait-il (plusieurs réponses possibles) **:**

		Oui	Non
405.	**A pied ?**	☐ 1	☐ 2
406.	**En voiture familiale ?**	☐ 1	☐ 2
407.	**En bus scolaire ?**	☐ 1	☐ 2
408.	**En métro ?**	☐ 1	☐ 2
409.	**En autobus ?**	☐ 1	☐ 2
410.	**En vélo ?**	☐ 1	☐ 2
411.	**En moto, scooter ?**	☐ 1	☐ 2
412.	**Autre ?**	☐ 1	☐ 2

Par ailleurs, le futur diabétique était-il transporté ou se déplaçait-il :

		Au moins une fois par semaine	Au moins une fois par mois	Plus rarement	Jamais
413.	**En métro ?**	☐ 1	☐ 2	☐ 3	☐ 4
414.	**En bus ?**	☐ 1	☐ 2	☐ 3	☐ 4

415. **Avait-il pris plusieurs fois le train ?** ☐ 1 Oui ☐ 2 Non

416. **Avait-il pris l'avion ?** ☐ 1 Oui ☐ 2 Non

417. **Le futur diabétique s'est-il rendu à l'étranger ?**

☐ 1 Oui ☐ 2 Non ⇨ *Allez à la question 418*

Si oui, dans quelle région :

		Oui	Non
417.1.	**Europe ?**	☐ 1	☐ 2
417.2.	**Afrique du nord ?**	☐ 1	☐ 2
417.3.	**Reste de l'Afrique ?**	☐ 1	☐ 2
417.4.	**Amérique du nord ?**	☐ 1	☐ 2
417.5.	**Amérique centrale ou Amérique du sud ?**	☐ 1	☐ 2
417.6.	**Asie ?**	☐ 1	☐ 2
417.7.	**Océanie ?**	☐ 1	☐ 2

418. **Avant le diabète, le patient était-il allé aux sports d'hiver ?** ☐ 1 Oui ☐ 2 Non

© 2008-2016, INSERM U 1169

419. **Avant le diabète, le patient était-il allé au bord de la mer ?**

☐ 1 Oui ☐ 2 Non ⇨ *Allez à la question 420*

419.1. **Si oui, combien a-t-il fait de séjours à la mer entre sa naissance et le diagnostic du diabète ?**

☐ 1 Un séjour
☐ 1 Entre un et cinq séjours
☐ 1 Plus de cinq séjours

**Le patient :**	Oui	Non
419.2. **Allait-il au bord de l'Atlantique ?**	☐ 1	☐ 2
419.3. **Allait-il au bord de la Méditerranée ?**	☐ 1	☐ 2
419.4. **Allait-il au bord d'une autre mer ?**	☐ 1	☐ 2
419.5. **Allait-il sur la plage ?**	☐ 1	☐ 2
419.6. **Se baignait-il dans la mer ?**	☐ 1	☐ 2

**Le patient :**	Oui	Non
420. **Allait-il à la piscine municipale ?**	☐ 1	☐ 2
421. **À la piscine chez des amis ?**	☐ 1	☐ 2
422. **Faisait-il régulièrement du poney ?**	☐ 1	☐ 2
423. **Participait-il régulièrement aux activités d'un club avec d'autres enfants (sports, musique...) ?**	☐ 1	☐ 2
424. **Jouait-il le week-end avec d'autres enfants ?**	☐ 1	☐ 2
425. **Était-il plutôt solitaire ?**	☐ 1	☐ 2

426. **Le patient était-il exposé au soleil ?**

☐ 1 Tous les jours
☐ 2 Au moins une fois par semaine
☐ 3 Au moins une fois par mois
☐ 4 Plus rarement
☐ 5 Pendant les vacances seulement
☐ 6 Jamais ou presque jamais

**Le patient faisait-il des promenades :**	Au moins une fois par jour	Au moins une fois par semaine	Au moins une fois par mois	Plus rarement	Jamais
427. **En ville ?**	☐ 1	☐ 2	☐ 3	☐ 4	☐ 5
428. **Au centre commercial ?**	☐ 1	☐ 2	☐ 3	☐ 4	☐ 5
429. **En forêt ?**	☐ 1	☐ 2	☐ 3	☐ 4	☐ 5
430. **Dans la campagne ?**	☐ 1	☐ 2	☐ 3	☐ 4	☐ 5
431. **En montagne ?**	☐ 1	☐ 2	☐ 3	☐ 4	☐ 5

432. **Le patient faisait-il des promenades au bord de rivières ?**

☐ 1 Au moins une fois par semaine
☐ 2 Au moins une fois par mois
☐ 3 Plus rarement

⇨ *Si vous avez coché une de ces cases, allez à la question 432.1*

☐ 4 Jamais ⇨ *Allez à la question 433*

432.1. **Le patient se baignait-il ou jouait-il dedans ?** ☐ 1 Oui ☐ 2 Non

433. **Le patient faisait-il des promenades au bord d'étangs, de lacs ou de mares ?**

433.1. ☐ 1 Oui **Si oui, se baignait-il ou jouait-il dedans ?** ☐ 1 Oui ☐ 2 Non

☐ 2 Non

© 2008-2016, INSERM U 1169

5.5 - Animaux des environs avant la découverte du diabète

434. **Le patient avait été au zoo ?**

☐ 1 Jamais
☐ 2 Une fois
☐ 3 Plusieurs fois

435. **Le patient avait-il été dans un magasin d'animaux (poissons, reptiles, oiseaux) ?**

☐ 1 Jamais
☐ 2 Une fois
☐ 3 Plusieurs fois

Animaux domestiques

436. **Au domicile familial, chez la nourrice ou dans un lieu fréquenté souvent par le patient (grands-parents, voisins, amis), y avait-il un ou des chiens ?**

☐ 1 Oui ☐ 2 Non ⇨ *Allez à la question 437*

436.1. **Si oui, combien ?** ☐ 1 Un chien ☐ 2 Plus d'un chien

	**De quelle race ?**	Oui	Non
436.2.	**«Bâtard» ?**	☐ 1	☐ 2
436.3.	**Labrador ?**	☐ 1	☐ 2
436.4.	**Caniche ?**	☐ 1	☐ 2
436.5.	**Yorkshire ?**	☐ 1	☐ 2
436.6.	**Berger allemand ?**	☐ 1	☐ 2
436.7.	**Epagneul breton ?**	☐ 1	☐ 2
436.8.	**Autre Berger ?**	☐ 1	☐ 2
436.9.	**Bichon ?**	☐ 1	☐ 2
436.10.	**Cocker ?**	☐ 1	☐ 2
436.11.	**Fox-Terrier ?**	☐ 1	☐ 2
436.12.	**Boxer/Shih-tzu ?**	☐ 1	☐ 2
436.13.	**Autre ?**	☐ 1	☐ 2

436.14. **Y avait-il eu des naissances de chiots ?** ☐ 1 Oui ☐ 2 Non

437. **Au domicile familial, chez la nourrice ou dans un lieu fréquenté souvent par le patient (grands-parents, voisins, amis), y avait-il des chats ?**

☐ 1 Oui ☐ 2 Non ⇨ *Allez à la question 438 page 43*

437.1. **Si oui, combien ?** ☐ 1 Un chat ☐ 2 Plus d'un chat

	**de quelle race ?**	Oui	Non
437.2.	**Européen (chats de gouttières) ?**	☐ 1	☐ 2
437.3.	**Siamois ?**	☐ 1	☐ 2
437.4.	**Persan ?**	☐ 1	☐ 2
437.5.	**Chartreux ?**	☐ 1	☐ 2
437.6.	**Abyssin/Somali ?**	☐ 1	☐ 2
437.7.	**Autres ?**	☐ 1	☐ 2

437.8. **Y avait-il eu des naissances de chatons ?** ☐ 1 Oui ☐ 2 Non

© 2008-2016, INSERM U 1169

Au domicile familial, chez la nourrice ou dans un lieu fréquenté souvent par le patient (grands-parents, voisins, amis), y avait-il :

		Oui	Non
438.	**Un petit rongeur (hamster, cochon d'inde...) ?**	☐ 1	☐ 2
439.	**Un lapin ?**	☐ 1	☐ 2
440.	**Un furet ?**	☐ 1	☐ 2
441.	**Des poissons d'aquarium d'eau douce ?**	☐ 1	☐ 2
442.	**Des poissons d'aquarium d'eau de mer ?**	☐ 1	☐ 2
443.	**Des poissons rouges dans un bassin accessible au patient ?**	☐ 1	☐ 2
444.	**Une tortue d'eau ?**	☐ 1	☐ 2
445.	**Une tortue terrestre ?**	☐ 1	☐ 2
446.	**Une poule ?**	☐ 1	☐ 2
447.	**D'autres volailles de basse-cour (canards, oies, pintades...) ?**	☐ 1	☐ 2
448.	**Une vache ?**	☐ 1	☐ 2
449.	**Des moutons, chèvres ?**	☐ 1	☐ 2
450.	**Un cochon ?**	☐ 1	☐ 2
451.	**Un cheval ou un poney ?**	☐ 1	☐ 2
452.	**Plusieurs chevaux ou poneys ?**	☐ 1	☐ 2

453. **Au domicile familial, chez la nourrice ou dans un lieu fréquenté souvent par le patient (grands-parents, voisins, amis), y avait-il des oiseaux en cage ?**

☐ 1 Oui ☐ 2 Non

454. **Au domicile familial, chez la nourrice ou dans un lieu fréquenté souvent par le patient (grands-parents, voisins, amis), y avait-il un autre animal familier au patient ?**

☐ 1 Oui ☐ 2 Non

La santé des animaux domestiques

455. **Un des animaux familiers du patient était-il tombé malade pendant la période considérée ?**

☐ 1 Oui ☐ 2 Non ⇨ *Allez à la question 456 page 44*

Si oui, quel animal (plusieurs réponses possibles) :

		Oui	Non
455.1.	**Un chat ?**	☐ 1	☐ 2
455.2.	**Un chien ?**	☐ 1	☐ 2
455.3.	**Un rongeur, lapin ?**	☐ 1	☐ 2
455.4.	**Un poisson ?**	☐ 1	☐ 2
455.5.	**Un oiseau ?**	☐ 1	☐ 2
455.6.	**Un autre animal ?**	☐ 1	☐ 2

Quelle était la cause de la maladie (plusieurs réponses possibles) :

		Oui	Non
455.7.	**Une infection ?**	☐ 1	☐ 2
455.8.	**Une paralysie ?**	☐ 1	☐ 2
455.9.	**Un diabète ?**	☐ 1	☐ 2
455.10.	**Un accident ?**	☐ 1	☐ 2
455.11.	**Inconnue ?**	☐ 1	☐ 2
455.12.	**Une autre cause ?**	☐ 1	☐ 2

© 2008-2016, INSERM U 1169

456. **Un des animaux familiers du patient était-il mort avant le diagnostic du diabète ?**

☐ 1 Oui ☐ 2 Non ⇨ *Allez à la question 457*

Si oui, quel animal (plusieurs réponses possibles) :

		Oui	Non
456.1.	**Un chat ?**	☐ 1	☐ 2
456.2.	**Un chien ?**	☐ 1	☐ 2
456.3.	**Un rongeur, lapin ?**	☐ 1	☐ 2
456.4.	**Un poisson ?**	☐ 1	☐ 2
456.5.	**Un oiseau ?**	☐ 1	☐ 2
456.6.	**Un autre animal ?**	☐ 1	☐ 2

Quelle était la cause de la mort (plusieurs réponses possibles) :

		Oui	Non
456.7.	**Une infection ?**	☐ 1	☐ 2
456.8.	**Une paralysie ?**	☐ 1	☐ 2
456.9.	**Un diabète ?**	☐ 1	☐ 2
456.10.	**Un accident ?**	☐ 1	☐ 2
456.11.	**La vieillesse ?**	☐ 1	☐ 2
456.12.	**Inconnue ?**	☐ 1	☐ 2
456.13.	**Une autre cause ?**	☐ 1	☐ 2

Les animaux du voisinage

Pensez aux animaux présents dans le proche voisinage (à moins de 100 mètres) : vaches, moutons, porcs, chevaux, etc

En dehors de son domicile (voisin, école, promenades), le patient avait-il eu une possibilité de contact direct avec :

		Oui assez souvent	Non exceptionellement
457.	**Un chien ?**	☐ 1	☐ 2
458.	**Un chat ?**	☐ 1	☐ 2
459.	**Un petit rongeur (hamster, cochon d'inde...) ?**	☐ 1	☐ 2
460.	**Un lapin ?**	☐ 1	☐ 2
461.	**Un furet ?**	☐ 1	☐ 2
462.	**Des poissons d'aquarium d'eau douce ?**	☐ 1	☐ 2
463.	**Des poissons d'aquarium d'eau de mer ?**	☐ 1	☐ 2
464.	**Des poissons rouges dans un bassin accessible au patient ?**	☐ 1	☐ 2
465.	**Une tortue d'eau ?**	☐ 1	☐ 2
466.	**Une tortue terrestre ?**	☐ 1	☐ 2
467.	**Des oiseaux en cage ?**	☐ 1	☐ 2
468.	**Des poules ?**	☐ 1	☐ 2
469.	**D'autres volailles de basse-cour (canards, oies, pintades...) ?**	☐ 1	☐ 2
470.	**Des pigeons ?**	☐ 1	☐ 2
471.	**Des canards sauvages ?**	☐ 1	☐ 2
472.	**Des cygnes ?**	☐ 1	☐ 2
473.	**Une vache ?**	☐ 1	☐ 2
474.	**Des moutons, chèvres ?**	☐ 1	☐ 2
475.	**Un cochon ?**	☐ 1	☐ 2
476.	**Un cheval ou poney ?**	☐ 1	☐ 2

© 2008-2016, INSERM U 1169

477. **Avant le diabète, le patient n'était en contact avec aucun animal ?** ☐ 1 Vrai ☐ 2 Faux

478. **Dans l'habitation ou le jardin, y avait-il des souris ?** ☐ 1 Oui ☐ 2 Non

Dans le voisinage de l'habitation (<u>moins de 200 mètres</u>) y avait-il :

	Oui	Non
479. **Des renards ?**	☐ 1	☐ 2
480. **Des hérissons ?**	☐ 1	☐ 2
481. **Des cervidés (chevreuils, cerfs) ?**	☐ 1	☐ 2
482. **Des pigeons (de ville ou ramiers) ?**	☐ 1	☐ 2
483. **Des mouettes ?**	☐ 1	☐ 2
484. **Des corbeaux ou corneilles ?**	☐ 1	☐ 2
485. **Des chauve-souris ?**	☐ 1	☐ 2
486. **Un étang ou une mare ?**	☐ 1	☐ 2
487. **Une rivière ou un canal ?**	☐ 1	☐ 2
488. **Un ruisseau ou une source ?**	☐ 1	☐ 2
489. **Une forêt ?**	☐ 1	☐ 2
490. **Une ferme ?**	☐ 1	☐ 2
491. **Des vaches ?**	☐ 1	☐ 2
492. **Des moutons, chèvres ?**	☐ 1	☐ 2
493. **Des cochons ?**	☐ 1	☐ 2
494. **Des volailles ?**	☐ 1	☐ 2
495. **Des chevaux ou poneys ?**	☐ 1	☐ 2

Les animaux pendant les vacances

Pendant les vacances, quels animaux étaient présents dans le proche voisinage (<u>moins de 100 mètres</u>) :

	Oui	Non
496. **Un chien ?**	☐ 1	☐ 2
497. **Un chat ?**	☐ 1	☐ 2
498. **De petits rongeurs : souris, rats, mulots... ?**	☐ 1	☐ 2
499. **Un lapin ?**	☐ 1	☐ 2
500. **Des poissons, tortues en aquarium ?**	☐ 1	☐ 2
501. **Des oiseaux en cage ?**	☐ 1	☐ 2
502. **Des poules ?**	☐ 1	☐ 2
503. **D'autres volailles de basse-cour : canards, oies, pintades... ?**	☐ 1	☐ 2
504. **Des pigeons ?**	☐ 1	☐ 2
505. **Une vache ?**	☐ 1	☐ 2
506. **Des moutons, des chèvres ?**	☐ 1	☐ 2
507. **Un cochon ?**	☐ 1	☐ 2
508. **Un cheval ou poney ?**	☐ 1	☐ 2
509. **D'autres animaux ?**	☐ 1	☐ 2

510. **En vacances, le patient n'était en contact avec aucun animal ?** ☐ 1 Vrai ☐ 2 Faux

© 2008-2016, INSERM U 1169

511. **Le père ou la mère étaient-ils en contact avec des animaux domestiques en dehors du domicile (chevaux, chiens, chats, etc) ?**

☐ 1 Oui ☐ 2 Non ⇨ *Allez à la question 512 page 47*

511.1. **Si oui, avec quelle fréquence ?**

☐ 1 Tous les jours
☐ 2 Au moins une fois par semaine
☐ 3 Au moins une fois par mois
☐ 4 Plus rarement

**De quels animaux s'agissait-il :**	Oui	Non
511.2. **Chiens ?**	☐ 1	☐ 2
511.3. **Chats ?**	☐ 1	☐ 2
511.4. **Poules, canards, oies... ?**	☐ 1	☐ 2
511.5. **Lapins ?**	☐ 1	☐ 2
511.6. **Ovins (chèvres, moutons) ?**	☐ 1	☐ 2
511.7. **Vaches ?**	☐ 1	☐ 2
511.8. **Chevaux ?**	☐ 1	☐ 2
511.9. **Autres ?**	☐ 1	☐ 2

© 2008-2016, INSERM U 1169

6 - Les parents et proches du futur diabétique

6.1 - La famille du patient avant la découverte du diabète

La santé et les proches

Attention ! Les questions qui suivent concernent la période allant de la naissance à la découverte du diabète

Les parents

512. **La mère fumait-elle, durant cette période ?**

512.1. ☐ 1 Oui **Si oui, comment ?**
☐ 1 Moins de 15 cigarettes par jour
☐ 2 Plus de 15 cigarettes par jour

512.2. **Fumait-elle en présence du patient ?** ☐ 1 Oui ☐ 2 Non

☐ 2 Non

513. **Le père fumait-il durant cette période ?**

513.1. ☐ 1 Oui **Si oui, comment ?**
☐ 1 Moins de 15 cigarettes par jour
☐ 2 Plus de 15 cigarettes par jour

513.2. **Fumait-il en présence du patient ?** ☐ 1 Oui ☐ 2 Non

☐ 2 Non

514. **Une autre personne fumait-elle régulièrement au domicile familial ?** ☐ 1 Oui ☐ 2 Non

515. **L'un ou l'autre des parents a-t-il reçu des soins dentaires durant cette période ?** ☐ 1 Oui ☐ 2 Non

Entre la naissance du patient et la découverte du diabète, l'un ou l'autre des parents a-t-il eu :

	Oui	Non
516. **La rougeole ?**	☐ 1	☐ 2
517. **Les oreillons ?**	☐ 1	☐ 2
518. **La varicelle ?**	☐ 1	☐ 2
519. **Un syndrome pied-main-bouche (vésicules sur les paumes, les plantes de pieds et dans la bouche) ?**	☐ 1	☐ 2
520. **La grippe ?**	☐ 1	☐ 2
521. **Une infection respiratoire ou une bronchiolite ?**	☐ 1	☐ 2
522. **Une hépatite ?**	☐ 1	☐ 2
523. **Des épisodes fébriles répétés ?**	☐ 1	☐ 2
524. **Une méningite virale ?**	☐ 1	☐ 2

525. **Un ou l'autre des parents a-t-il eu une diarrhée aiguë avec des vomissements ?**

525.1. ☐ 1 Oui **Si oui, d'été ?** ☐ 1 Oui ☐ 2 Non

525.2. **d'hiver ?** ☐ 1 Oui ☐ 2 Non

☐ 2 Non

© 2008-2016, INSERM U 1169

Entre la naissance du patient et le diagnostic du diabète, <u>la mère</u> a-t-elle pris :

	souvent<br>5 fois par an ou plus souvent	assez souvent<br>3 à 4 fois par an	rarement<br>1 ou 2 fois par an	Jamais
526. **Du paracétamol** (Doliprane, Efferalgan, Dafalgan...)**, de l'ibuprofen** (Advyl, Nurofen...) **ou de l'aspirine** (Aspegic...)......................	☐ 1	☐ 2	☐ 3	☐ 4
527. **Un antiviral :** Zovirax	☐ 1	☐ 2	☐ 3	☐ 4
528. **Un anti-inflammatoire stéroïdien :** Solupred, Celestene, Cortancyl...	☐ 1	☐ 2	☐ 3	☐ 4
529. **Un antibiotique :** Amoxicilline, Clamoxil, Orelox, Augmentin, Zithromax...	☐ 1	☐ 2	☐ 3	☐ 4
530. **Un médicament contre les allergies :** Atarax, Polaramine, Zyrtec, Cétirizine, Aerius, Clarityne...	☐ 1	☐ 2	☐ 3	☐ 4
531. **Un médicament contre le cholestérol :** Crestor, Tahor, Zocor...	☐ 1	☐ 2	☐ 3	☐ 4
532. **Un médicament contre la toux :** Toplexil, Néocodion...	☐ 1	☐ 2	☐ 3	☐ 4
533. **Un bronchodilatateur :** Ventoline, Serevent...	☐ 1	☐ 2	☐ 3	☐ 4
534. **Un médicament contre les brûlures d'estomac, l'acidité gastrique :** Maalox, Mopral (Omeprazol).	☐ 1	☐ 2	☐ 3	☐ 4
535. **Un antidiarrhéique :** Ultralevure, Lactéol	☐ 1	☐ 2	☐ 3	☐ 4
536. **Un antidiarrhéique :** Imodium, Tiorfan...	☐ 1	☐ 2	☐ 3	☐ 4
537. **Un médicament contre la constipation :** Transipeg, Spagulax, Forlax, Lactulose...	☐ 1	☐ 2	☐ 3	☐ 4

Entre la naissance du patient et le diagnostic du diabète, <u>le père</u> a-t-il pris :

	souvent<br>5 fois par an ou plus souvent	assez souvent<br>3 à 4 fois par an	rarement<br>1 ou 2 fois par an	Jamais
538. **Du paracétamol** (Doliprane, Efferalgan, Dafalgan...)**, de l'ibuprofen** (Advyl, Nurofen...) **ou de l'aspirine** (Aspegic...)......................	☐ 1	☐ 2	☐ 3	☐ 4
539. **Un antiviral :** Zovirax	☐ 1	☐ 2	☐ 3	☐ 4
540. **Un anti-inflammatoire stéroïdien :** Solupred, Celestene, Cortancyl...	☐ 1	☐ 2	☐ 3	☐ 4
541. **Un antibiotique :** Amoxicilline, Clamoxil, Orelox, Augmentin, Zithromax...	☐ 1	☐ 2	☐ 3	☐ 4
542. **Un médicament contre les allergies :** Atarax, Polaramine, Zyrtec, Cétirizine, Aerius, Clarityne...	☐ 1	☐ 2	☐ 3	☐ 4
543. **Un médicament contre le cholestérol :** Crestor, Tahor, Zocor...	☐ 1	☐ 2	☐ 3	☐ 4
544. **Un médicament contre la toux :** Toplexil, Néocodion...	☐ 1	☐ 2	☐ 3	☐ 4
545. **Un bronchodilatateur :** Ventoline, Serevent...	☐ 1	☐ 2	☐ 3	☐ 4
546. **Un médicament contre les brûlures d'estomac, l'acidité gastrique :** Maalox, Mopral (Omeprazol).	☐ 1	☐ 2	☐ 3	☐ 4
547. **Un antidiarrhéique :** Ultralevure, Lactéol	☐ 1	☐ 2	☐ 3	☐ 4
548. **Un antidiarrhéique :** Imodium, Tiorfan...	☐ 1	☐ 2	☐ 3	☐ 4

© 2008-2016, INSERM U 1169

**Le père a-t-il :**	Oui	Non
549. **Une maladie cardio-vasculaire ?**	☐ 1	☐ 2
550. **Un excès de cholestérol ?**	☐ 1	☐ 2
551. **De l'hypertension artérielle ?**	☐ 1	☐ 2
552. **Un diabète de type 1 ?**	☐ 1	☐ 2
553. **Un diabète de type 2 ?**	☐ 1	☐ 2

554. **Un des parents a-t-il fait un ou plusieurs séjours à l'étranger pendant la petite enfance du futur diabétique ?**

☐ 1 Oui ☐ 2 Non ⇨ *Allez à la question 555*

**Si oui, dans quelle région :**	Oui	Non
554.1. **Europe ?**	☐ 1	☐ 2
554.2. **Afrique du nord ?**	☐ 1	☐ 2
554.3. **Reste de l'Afrique ?**	☐ 1	☐ 2
554.4. **Amérique du nord ?**	☐ 1	☐ 2
554.5. **Amérique centrale ou Amérique du sud ?**	☐ 1	☐ 2
554.6. **Asie ?**	☐ 1	☐ 2

Les frères et sœurs

555. **Un frère ou une sœur du patient porte-t-il des lunettes ou/et des lentilles de correction de vue?**

555.1. ☐ 1 Oui

☐ 2 Non

556. **Un frère ou une sœur du témoin est-il myope ?** ☐ 1 Oui ☐ 2 Non

557. **Un frère ou une sœur a-t-il un diabète ?** ☐ 1 Oui ☐ 2 Non

Les questions qui suivent concernent les frères ou sœurs du patient entre sa naissance et la découverte de son diabète

558. **Durant cette période le patient avait-il des frères et sœurs vivant dans le foyer ?**

☐ 1 Oui ☐ 2 Non ⇨ *Allez à la question 559 page 50*

Sans toutefois vous livrer à une enquête trop poussée, pouvez-vous porter une attention particulière aux questions suivantes ?

**Si oui, un frère ou une sœur a-t-il eu :**	Oui	Non
558.1. **Un syndrome pied-main-bouche (vésicules sur les paumes, les plantes de pieds et dans la bouche) ?**	☐ 1	☐ 2
558.2. **Une infection respiratoire ou une bronchiolite ?**	☐ 1	☐ 2
558.3. **Une hépatite ?**	☐ 1	☐ 2
558.4. **Des épisodes fébriles répétés ?**	☐ 1	☐ 2
558.5. **Une méningite virale ?**	☐ 1	☐ 2

558.6. **Un frère ou une sœur a-t-il eu une diarrhée aiguë avec des vomissements ?**

558.6.1. ☐ 1 Oui **Si oui, d'été ?** ☐ 1 Oui **d'hiver ?** ☐ 2 Oui

☐ 2 Non

© 2008-2016, INSERM U 1169

558.7. **Les frères et sœurs ont-ils eu une maladie infectieuse sans gravité, mais avec de la fièvre (rhinopharyngites, grippes, otites, gastro-entérites) ?**

☐ 1 Rarement
☐ 2 Parfois
☐ 3 Assez souvent
☐ 4 Souvent
☐ 5 Très souvent

558.8. **En quelle saison (plusieurs réponses possibles) :**

☐ 1 Au printemps ☐ 2 En été ☐ 3 En automne ☐ 4 En hiver

558.9. **Un frère ou une sœur a-t-il un diabète ?** ☐ 1 Oui ☐ 2 Non

Les grands-parents et les autres proches de la famille avec lesquels le patient a été en contact

559. **Entre sa naissance et le diagnostic du diabète, le patient était-il en contact avec l'un au moins de ses grands-parents :**

☐ 1 Tous les jours
☐ 2 Au moins une fois par semaine
☐ 3 Au moins une fois par mois
☐ 4 Plus rarement
☐ 5 Jamais

Sans toutefois vous livrer à une enquête trop poussée,
pouvez-vous porter une attention particulière aux questions suivantes ?

**Un des grands-parents ou un des proches de la famille a-t-il eu :**	Oui	Non
560. **Un syndrome pied-main-bouche (éruption des paumes et des plantes et bulles dans la bouche) ?**	☐ 1	☐ 2
561. **La rougeole ?**	☐ 1	☐ 2
562. **La varicelle ?**	☐ 1	☐ 2
563. **Les oreillons ?**	☐ 1	☐ 2
564. **Une infection respiratoire ou une bronchiolite ?**	☐ 1	☐ 2
565. **La grippe ?**	☐ 1	☐ 2
566. **Une diarrhée aiguë avec des vomissements ?**	☐ 1	☐ 2
567. **Une hépatite ?**	☐ 1	☐ 2
568. **Des épisodes fébriles répétés ?**	☐ 1	☐ 2
569. **Une méningite virale ?**	☐ 1	☐ 2

6.2 - Loisirs des parents avant la découverte du diabète

Ces questions ne concernent que les loisirs des parents
en dehors de la présence du patient diabétique

570. **Le père ou la mère faisaient à cette période des randonnées ou du jogging en forêt :**

☐ 1 Au moins une fois par semaine
☐ 2 Au moins une fois par mois
☐ 3 Plus rarement
☐ 4 Jamais

© 2008-2016, INSERM U 1169

571. **Le père ou la mère faisaient-ils des promenades ou du jogging dans la campagne :**

- ☐ 1 Au moins une fois par semaine
- ☐ 2 Au moins une fois par mois
- ☐ 3 Plus rarement
- ☐ 4 Jamais

**Le père ou la mère :**	Oui	Non
572. **Jardinaient-ils (fleurs, plantes d'ornement) ?**	☐ 1	☐ 2
573. **Jardinaient-ils (légumes, potager) ?**	☐ 1	☐ 2
574. **Cueillaient-ils des champignons sauvages ?**	☐ 1	☐ 2

575. **Le père ou la mère étaient-ils chasseurs?** ☐ 1 Oui ☐ 2 Non ⇨ *Allez à la question 576*

575.1. **Etait-ce avec leur(s) propre(s) chien(s) ?** ☐ 1 Oui ☐ 2 Non

575.2. **Le père ou la mère chassaient-ils:**

- ☐ 1 Moins d'une fois par trimestre
- ☐ 1 5 à 10 fois par an
- ☐ 1 Plus souvent

575.3. **Ramenaient-ils du gibier à la maison ?** ☐ 1 Oui ☐ 2 Non ⇨ *Allez à la question 576*

**Si oui, de quel(s) animaux s'agissait-il :**	Oui	Non
575.3.1. **Sanglier ?**	☐ 1	☐ 2
575.3.2. **Cerf ?**	☐ 1	☐ 2
575.3.3. **Chevreuil ?**	☐ 1	☐ 2
575.3.4. **Lièvre ?**	☐ 1	☐ 2
575.3.5. **Canard et gibier d'eau ?**	☐ 1	☐ 2
575.3.6. **Lapin de garenne ?**	☐ 1	☐ 2
575.3.7. **Faisan ?**	☐ 1	☐ 2
575.3.8. **Perdreau ou perdrix ?**	☐ 1	☐ 2
575.3.9. **Autres ?**	☐ 1	☐ 2

575.3.10. **Le gibier était-il préparé et consommé en famille ?** ☐ 1 Oui ☐ 2 Non

576. **Le père ou la mère étaient-ils pêcheurs ?** ☐ 1 Oui ☐ 2 Non ⇨ *Le questionnaire est terminé, Merci !*

576.1. **En mer ?** ☐ 1 Oui ☐ 2 Non

576.2. **En eau douce ?** ☐ 1 Oui ☐ 2 Non

576.3. **Ramenaient-ils leur pêche à la maison ?** ☐ 1 Oui ☐ 2 Non ⇨ *Le questionnaire est terminé !*

**Si oui, de quel(s) animaux s'agissait-il :**	Oui	Non
576.3.1. **Truites ?**	☐ 1	☐ 2
576.3.2. **Brochets ?**	☐ 1	☐ 2
576.3.3. **Carpes ?**	☐ 1	☐ 2
576.3.4. **Autres ?**	☐ 1	☐ 2

576.3.5. **La pêche était-elle consommée en famille ?** ☐ 1 Oui ☐ 2 Non

Le questionnaire est terminé !
Merci de nous le faire parvenir dans l'enveloppe pré-affranchie.

© 2008-2016, INSERM U 1169

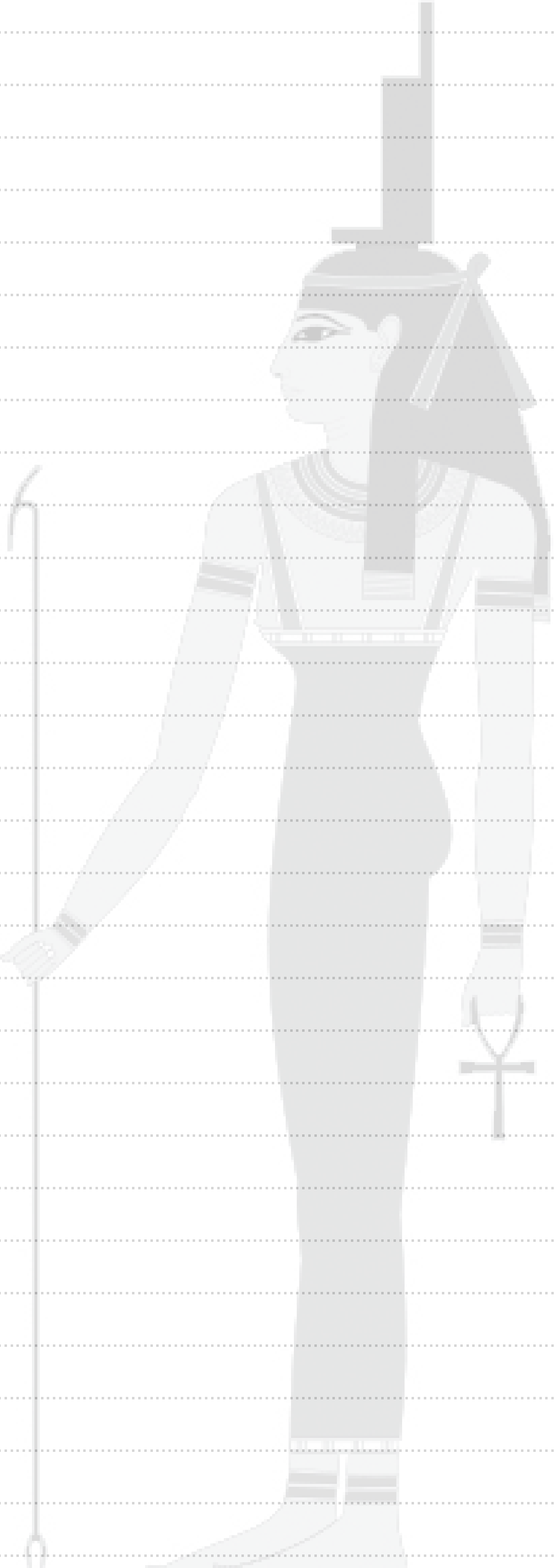

© 2008-2016, INSERM U 1169

© 2008-2016, INSERM U 1169

© 2008-2016, INSERM U 1169

© 2008-2016, INSERM U 1169

© 2008-2016, INSERM U 1169
