# Supplementary material for: Association of environmental markers with childhood type 1 diabetes mellitus revealed by a long questionnaire on early life exposures and lifestyle in a case–control study
Source: BMC Public Health. 2016 Sep 29;16:1021. doi: 10.1186/s12889-016-3690-9 (PMC5041527; doi:10.1186/s12889-016-3690-9)
Supplement: Additional file 3: — Additional analysis supporting the exclusion for primary school mistake and exploring further age-related bias. (PDF 5474 kb) [file 12889_2016_3690_MOESM3_ESM.pdf]

Supplementary material

I The primary school mistake

A first analysis of the data showed a significant association between primary school attendance and disease. Patients and controls were expected to be matched on age. A closer look at the data showed that a fraction of participants, especially controls, reported attendance to primary school before 6 years old. Admission in primary school is rarely allowed before 6 years old in France so it seemed like mistakes. We interpreted this mistake to be a marker that the whole questionnaire had been filled without respect to the reference age. In the main text, we excluded those participants where the mistake occurred. To assess if the primary school mistake was a one time error or if it impacted the rest of the questionnaire, we trained a random Forest on the questionnaire to predict reference age. Prediction results show that the exclusion was justified.

Prediction model for age

We trained a random Forest [1 p587-604] to predict age using the questionnaire. The rationale is that as a participant advances in age, he experiences more diverse environments. This allows to try and predict age using the answers to the questionnaire. While this prediction is by nature approximate, large differences between reference age and predicted age are suspected to reveal inadequate filling of the questionnaire.

We trained our model on the dataset obtained after the primary school mistake. We excluded the question regarding primary school from our model as we wanted to know if the rest of the questionnaire was influenced by a mistake on that question. We also excluded the 295 variables with more than 5% of missing data. After the exclusions, the overall missing rate was 2%. We performed a simple imputation of the remaining missing data using the na.roughfix function of the randomForest package [2]. We then trained a random Forest regression on the completed dataset. The default parameters for regression were used.

The trained random Forest out-of bag estimate accounted for 63% of the reference age variance in the training set. It did not predict ages above 12. This is understandable since the questionnaire is centered on early childhood and therefore no questions allow to distinguish a 15 years old from a 12 years old. We then used the model to predict the age of those who had wrongly answered the primary school question. Supplementary figure 1 justifies the exclusion of the 14 cases (1%) and 83 controls (10%) who made the primary school mistake as their predicted age is in general much larger than their reference age.

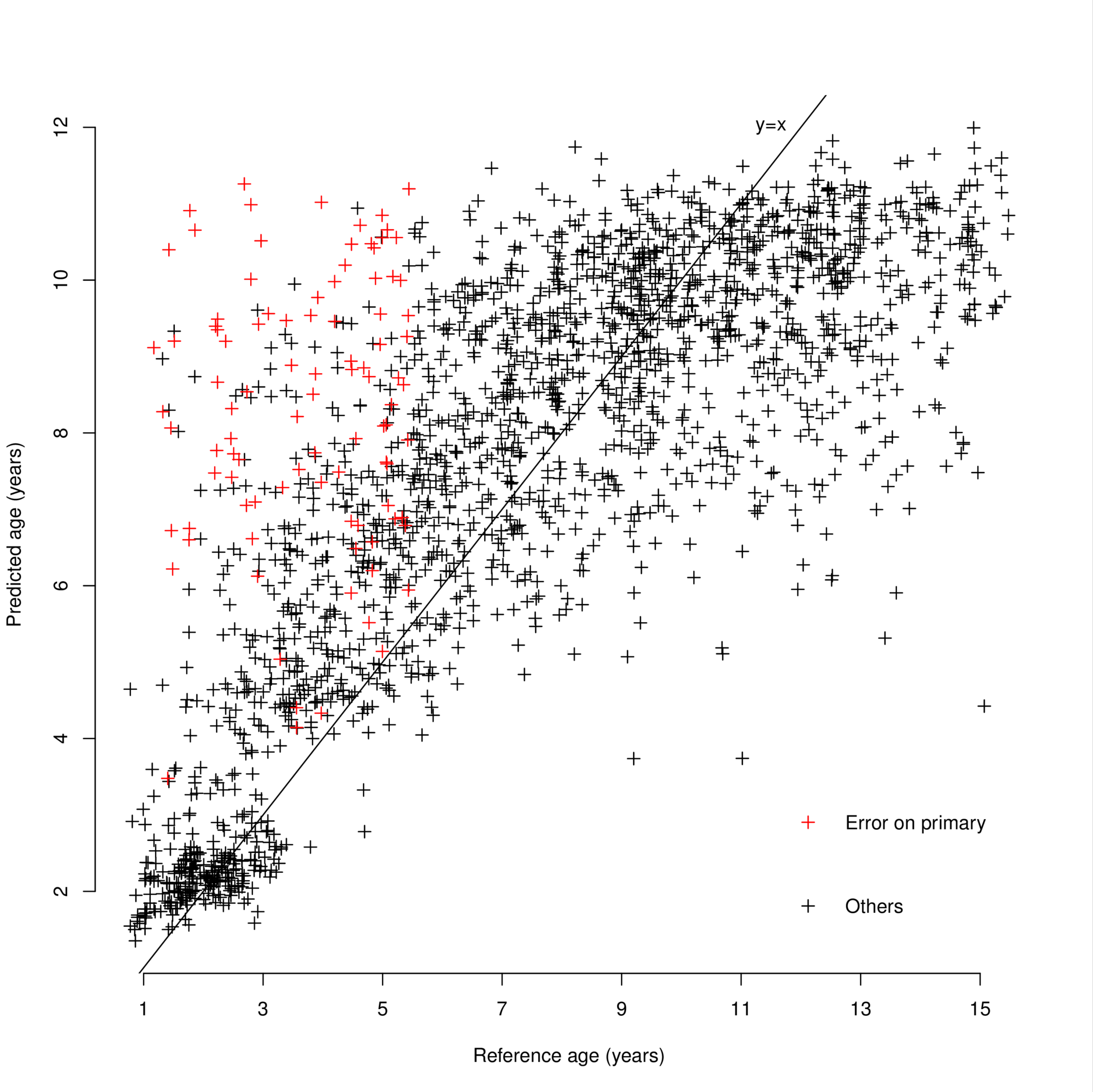

Supplementary figure 1: Predicted age for primary school mistake participants. The predicted age is plotted against the reference age. The predicted age is the out-of-bag estimate for the participants who did not make the primary school mistake (in black). The predicted age is the prediction of the entire forest for the ones who made the primary school mistake (in red). The line y=x corresponds to a perfect prediction.

II Additional age-related exclusion

In this section, using the same prediction model for age as above, we consider a further exclusion of participants whose predicted age is much larger than their reference age but did not make the primary school mistake. The excluded participants are again disproportionately controls. This exclusion is not as justified as there is no way to know if a mistake has indeed happened or if the participant simply had many experiences early in life. We study how the results are impacted by this further exclusion.

In the remaining participants, 16 patients (1%) and 39 controls (5%) had a difference between predicted age and reference age larger than 4 years. While those participants did not make the primary school mistake, we considered the possibility that they nevertheless filled the questionnaire

not paying much attention to the reference age. This is supported by the disproportion between cases and controls in this set. However, there is no definite way of knowing if there has been a mistake or not in this case and this is why we considered this exclusion only in the supplementary material. Supplementary figure 2 shows the set of excluded participants.

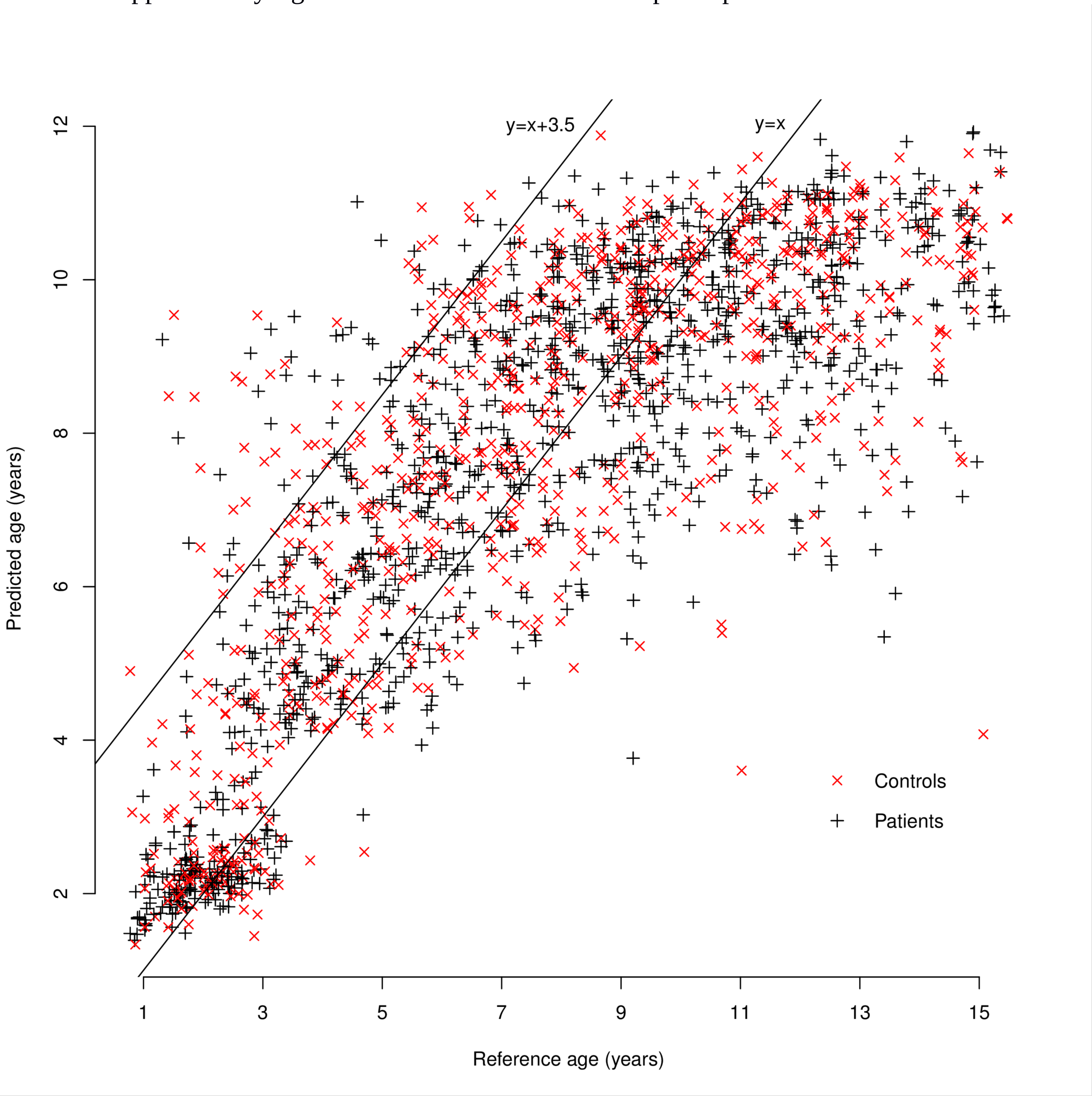

Supplementary figure 2: Predicted age much larger than reference in a subset. The out-of-bag estimate of age is plotted against the reference age. The line y=x corresponds to a perfect prediction. Over the line y=x+4, the participants are excluded.

We then defined once again two datasets in the same way as in the main text. The modified exclusion process is summarized in the following flowchart. We refer to the resulting datasets as modified. The datasets defined in the main text are referred to as original.

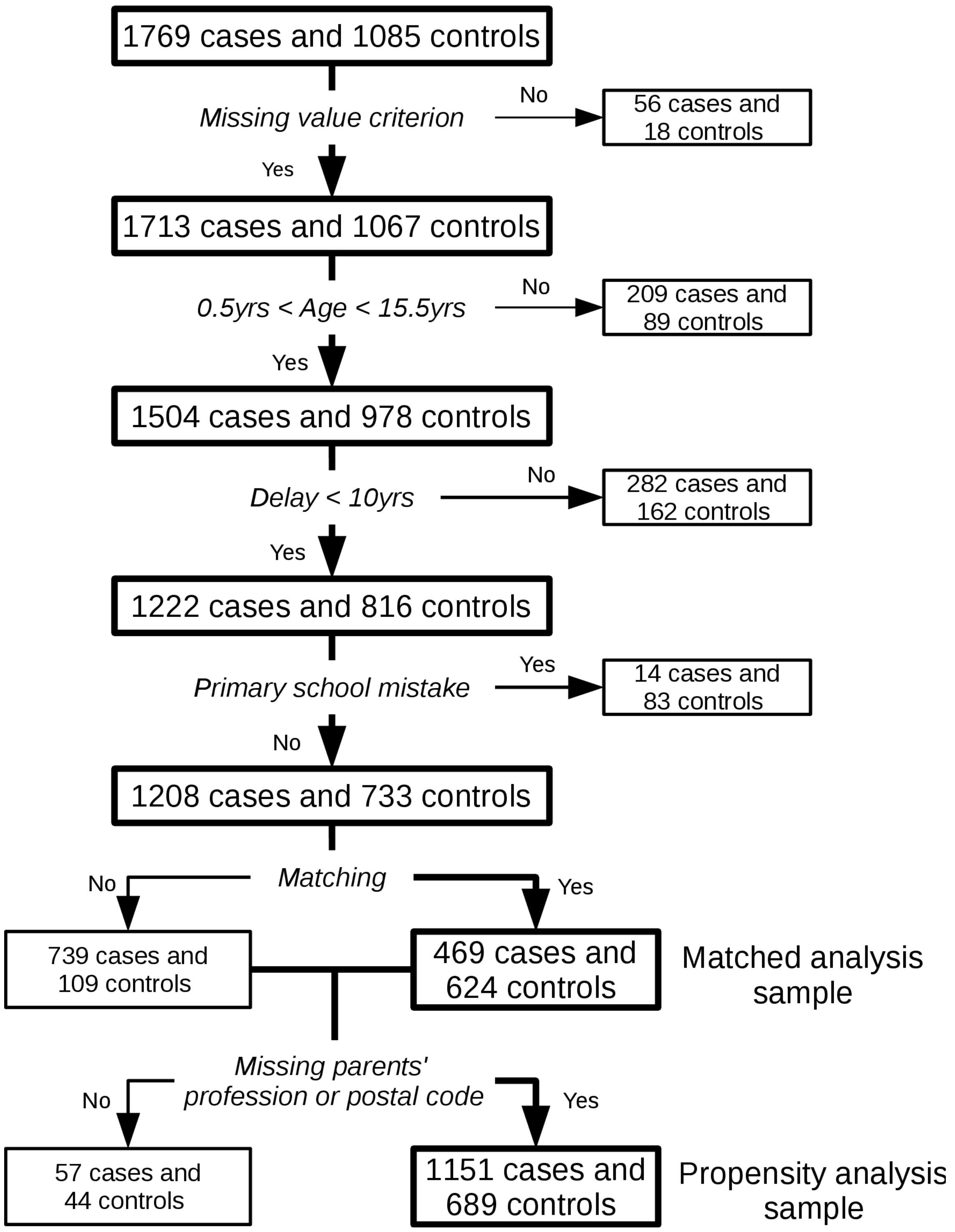

Supplementary flowchart of modified exclusions and sample definition.

Analysis of the modified datasets

On the list of the original significant results, we performed the same analysis as described in the main text in the modified datasets to evaluate the impact of the exclusion.

In order to determine if the drop in significance was simply due to smaller sample size, we defined an empirical distribution of p-values under random exclusion. We randomly select a subsample of the corresponding original dataset (matched or propensity) with the same sample size as the modified dataset. As missing data does not influence the results of the tests, for each variable, we exclude the missing data before determining the sample size. For the propensity analysis, having the same sample size means having the same number of patients and the same number of controls. For the matched analysis, having the same sample size means having the same number of strata of the

same type: one patient and one control or one patient and two controls. In this random subsample, we then perform the same analysis as in the main text. This gives a p-value. We then repeat this process 10000 times to obtain an empirical distribution of p-value under random subsampling. This allows us to test if the new p-value obtained for the modified dataset can be attributed to smaller sample size or not. The p-value of that test is then the proportion of the distribution that has a larger p-value than the modified p-value.

Results

The results are available in supplementary file 4. The exclusion affected significantly the two dentist variables and the beach variable for both analysis. The exclusion affected significantly for the propensity analysis the dental hygiene variable and the club variable. The drop in p-value is shown in supplementary figure 3. The significance of the drop compared to random exclusion for both p-values is shown in supplementary table 1.

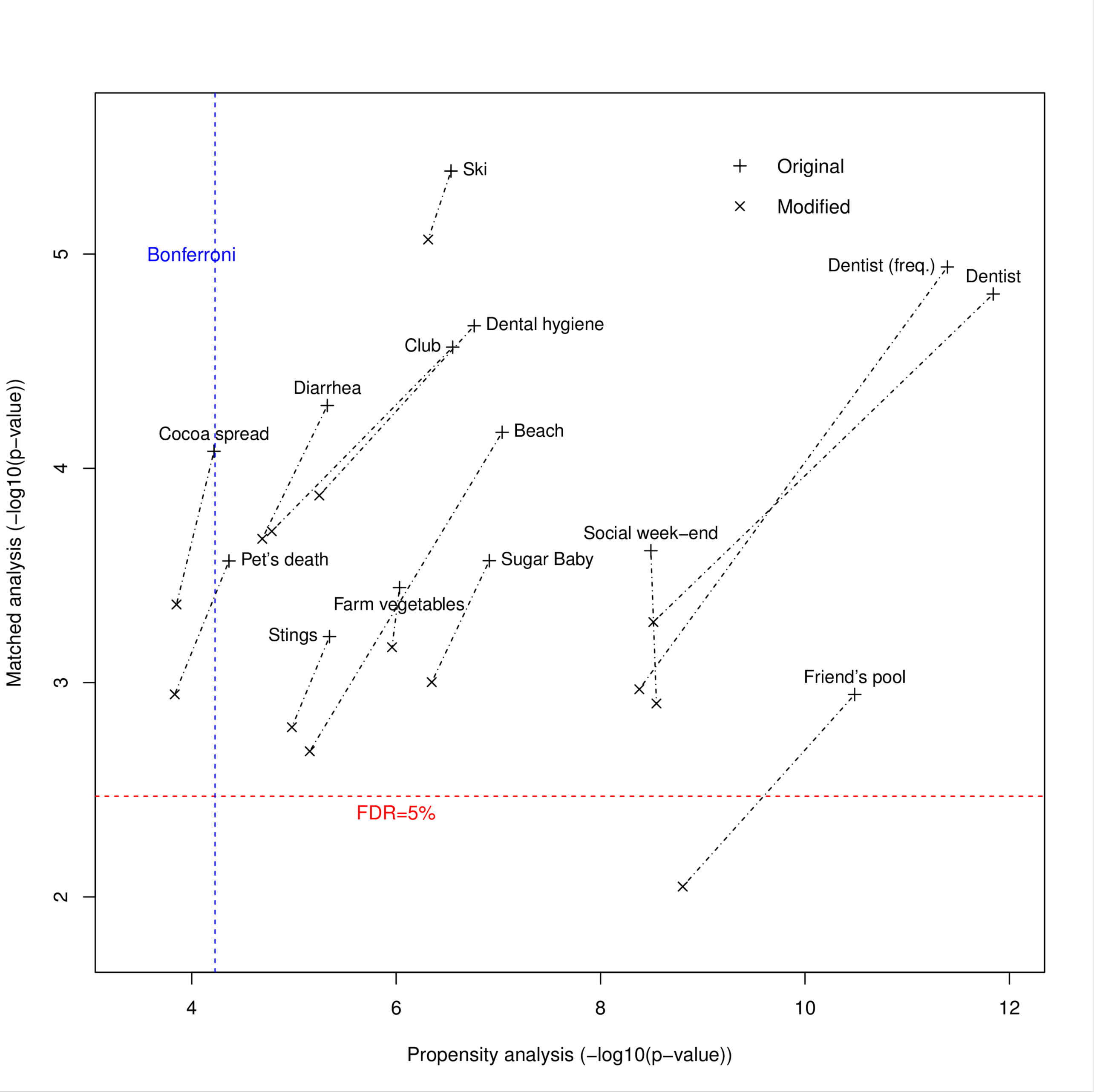

Supplementary figure 3: Drop of p-values after the exclusion. The red and blue line are the same threshold as in figure 4 of the main text. The original and modified p-value of the same variable are connected by a dotted line.

Label	P-value (Matched)	P-value (Propensity)
Sugar baby	0.2175	0.1801
Dentist	0.0013	0
Dentist (freq.)	0	0
Dental hygiene	0.1209	0.0028
Diarrhea	0.1599	0.0867
Stings	0.2477	0.3417
Farm vegetables	0.4555	0.7016
Cocoa spread	0.1026	0.2002
Ski	0.4433	0.2292
Beach	0.0002	0.0042
Friend's	0.0112	0.0611
Club	0.0664	0.0003
Social week-end	0.0876	0.729
Pet's death	0.1316	0.124

Supplementary table 1: Significance of the drop in p-value compared to a random exclusion. These p-values are obtained by subsampling 10000 times the original datasets. A p-value of 0 means that no occurence of the subsampling resulted in a p-value as large a s that obtained by the exclusion.

1. Hastie T, Tibshirani R, Friedman J (2009) The Elements of Statistical Learning. Springer New York, New York, NY

2. Liaw A, Wiener M (2002) Classification and Regression by randomForest. R News 2:18–22.
